# Supplementary material for: The Impact of Eucalyptus and Pine Plantations on the Taxonomic and Functional Diversity of Dung Beetles (Coleoptera: Scarabaeidae) in the Southern Region of Ecuador
Source: Biology (Basel). 2024 Oct 19;13(10):841. doi: 10.3390/biology13100841 (PMC11504286; doi:10.3390/biology13100841)
Supplement: Supplementary file 1 [file biology-13-00841-s001.zip › biology-3218356-supplementary.pdf]

Table S1. Abundance of dung beetles sampled per species, tramp, transect and land use

Plot_ID	Land use	Transect	Tramp	Species	Abundance
FL2	Forest	0	T1	Uroxys_sp2	12
FL2	Forest	0	T2	Onthophagus_curvicornis	2
FL2	Forest	150	T1	Uroxys_sp1	7
FL2	Forest	150	T1	Uroxys_sp2	3
FL2	Forest	150	T1	Ateuchus_aenomicans	18
FL2	Forest	150	T2	Onthophagus_curvicornis	11
FL2	Forest	150	T3	Onthophagus_curvicornis	1
FL2	Forest	150	T3	Uroxys_sp1	1
FL2	Forest	300	T1	Uroxys_sp1	1
FL2	Forest	300	T1	Uroxys_sp2	1
FL2	Forest	300	T2	Onthophagus_curvicornis	4
FL2	Forest	300	T2	Uroxys_sp1	1
FL2	Forest	300	T2	Dichotomius_cotopaxi	1
FL2	Forest	300	T3	Ateuchus_aenomicans	2
FL2	Forest	300	T3	Dichotomius_cotopaxi	1
FL1	Forest	0	T1	Dichotomius_cotopaxi	1
FL1	Forest	0	T2	Cryptocanthon_paradoxus	2
FL1	Forest	0	T2	Uroxys_sp2	6
FL1	Forest	0	T3	Dichotomius_cotopaxi	2
FL1	Forest	0	T3	Uroxys_sp2	4
FL1	Forest	150	T1	Dichotomius_cotopaxi	1
FL1	Forest	150	T1	Uroxys_sp2	25
FL1	Forest	150	T2	Uroxys_sp1	1
FL1	Forest	150	T2	Uroxys_sp2	3
FL1	Forest	150	T2	Cryptocanthon_paradoxus	14
FL1	Forest	150	T3	Uroxys_sp1	3
FL1	Forest	150	T3	Uroxys_sp2	35
FL1	Forest	300	T1	Uroxys_sp2	55
FL1	Forest	300	T2	Cryptocanthon_paradoxus	1
FL1	Forest	300	T3	Uroxys_sp1	3
FL1	Forest	300	T3	Uroxys_sp2	26
FL1	Forest	300	T3	Cryptocanthon_paradoxus	1
PL1	Pinus	0	T1	Onthophagus_curvicornis	6
PL1	Pinus	0	T1	Uroxys_sp2	68
PL1	Pinus	0	T2	Onthophagus_curvicornis	2
PL1	Pinus	0	T2	Uroxys_sp1	2
PL1	Pinus	0	T2	Uroxys_sp2	37
PL1	Pinus	0	T3	Onthophagus_curvicornis	1
PL1	Pinus	0	T3	Uroxys_sp1	21
PL1	Pinus	0	T3	Uroxys_sp2	172

PL1	Pinus	150	T1	Onthophagus_curvicornis	2
PL1	Pinus	150	T1	Uroxys_sp2	142
PL1	Pinus	150	T2	Uroxys_sp1	1
PL1	Pinus	150	T2	Uroxys_sp2	53
PL1	Pinus	150	T3	Uroxys_sp1	6
PL1	Pinus	150	T3	Uroxys_sp2	76
PL1	Pinus	150	T3	Onthophagus_curvicornis	1
PL1	Pinus	300	T1	Uroxys_sp1	5
PL1	Pinus	300	T1	Uroxys_sp2	36
PL1	Pinus	300	T1	Dichotomius_cotopaxi	1
PL1	Pinus	300	T1	Onthophagus_curvicornis	11
PL1	Pinus	300	T2	Uroxys_sp1	3
PL1	Pinus	300	T2	Uroxys_sp2	81
PL1	Pinus	300	T3	Uroxys_sp1	1
PL1	Pinus	300	T3	Uroxys_sp2	23
PL1	Pinus	300	T3	Onthophagus_curvicornis	6
PL2	Pinus	0	T2	Uroxys_sp1	1
PL2	Pinus	0	T2	Uroxys_sp2	5
PL2	Pinus	0	T2	Onthophagus_curvicornis	2
PL2	Pinus	0	T3	Uroxys_sp1	67
PL2	Pinus	0	T3	Uroxys_sp2	1
PL2	Pinus	0	T3	Dichotomius_cotopaxi	1
PL2	Pinus	0	T3	Onoreidium_aff_cristatum	1
PL2	Pinus	0	T3	Onthophagus_curvicornis	3
PL2	Pinus	150	T1	Onthophagus_curvicornis	3
PL2	Pinus	150	T2	Onthophagus_curvicornis	5
PL2	Pinus	150	T2	Uroxys_sp2	2
PL2	Pinus	150	T3	Uroxys_sp1	38
PL2	Pinus	150	T3	Uroxys_sp2	6
PL2	Pinus	150	T3	Dichotomius_cotopaxi	1
PL2	Pinus	150	T3	Onthophagus_curvicornis	8
PL2	Pinus	300	T1	Onthophagus_curvicornis	6
PL2	Pinus	300	T1	Uroxys_sp2	4
PL2	Pinus	300	T2	Uroxys_sp1	1
PL2	Pinus	300	T2	Uroxys_sp2	4
PL2	Pinus	300	T2	Dichotomius_cotopaxi	1
PL2	Pinus	300	T2	Onthophagus_curvicornis	13
PL2	Pinus	300	T3	Uroxys_sp1	7
PL2	Pinus	300	T3	Uroxys_sp2	13
PL2	Pinus	300	T3	Deltochilum_robustus	2
PL2	Pinus	300	T3	Dichotomius_cotopaxi	1
PL2	Pinus	300	T3	Onthophagus_curvicornis	3
EL2	Eucalyptus	0	T1	Uroxys_sp1	3

EL2	Eucalyptus	0	T1	Uroxys_sp2	1
EL2	Eucalyptus	0	T1	Onthophagus_curvicornis	4
EL2	Eucalyptus	0	T2	Onthophagus_curvicornis	8
EL2	Eucalyptus	0	T2	Uroxys_sp2	2
EL2	Eucalyptus	0	T2	Onoreidium_aff_cristatum	1
EL2	Eucalyptus	0	T3	Onthophagus_curvicornis	11
EL2	Eucalyptus	0	T3	Uroxys_sp2	2
EL2	Eucalyptus	150	T1	Onthophagus_curvicornis	4
EL2	Eucalyptus	150	T1	Uroxys_sp2	7
EL2	Eucalyptus	150	T2	Uroxys_sp1	2
EL2	Eucalyptus	150	T2	Onthophagus_curvicornis	3
EL2	Eucalyptus	150	T3	Onthophagus_curvicornis	9
EL2	Eucalyptus	150	T3	Uroxys_sp2	6
EL2	Eucalyptus	150	T3	Dichotomius_cotopaxi	1
EL2	Eucalyptus	300	T1	Uroxys_sp1	2
EL2	Eucalyptus	300	T1	Uroxys_sp2	84
EL2	Eucalyptus	300	T1	Onthophagus_curvicornis	1
EL2	Eucalyptus	300	T2	Onthophagus_curvicornis	3
EL2	Eucalyptus	300	T2	Uroxys_sp2	27
EL2	Eucalyptus	300	T3	Uroxys_sp1	2
EL2	Eucalyptus	300	T3	Uroxys_sp2	18
EL2	Eucalyptus	300	T3	Onthophagus_curvicornis	9
EL1	Eucalyptus	0	T1	Onthophagus_curvicornis	5
EL1	Eucalyptus	0	T2	Onthophagus_curvicornis	3
EL1	Eucalyptus	0	T3	Onthophagus_curvicornis	3
EL1	Eucalyptus	150	T1	Onthophagus_curvicornis	6
EL1	Eucalyptus	150	T2	Onthophagus_curvicornis	4
EL1	Eucalyptus	150	T3	Onthophagus_curvicornis	6
EL1	Eucalyptus	300	T1	Onthophagus_curvicornis	3
EL1	Eucalyptus	300	T1	Uroxys_sp2	2
EL1	Eucalyptus	300	T2	Onthophagus_curvicornis	4
EL1	Eucalyptus	300	T3	Onthophagus_curvicornis	4
FS1	Forest	0	T1	Homocropis_buckleyi	7
FS1	Forest	0	T2	Homocropis_buckleyi	4
FS1	Forest	0	T3	Homocropis_buckleyi	4
FS1	Forest	150	T1	Homocropis_buckleyi	7
FS1	Forest	150	T2	Homocropis_buckleyi	15
FS1	Forest	150	T3	Homocropis_buckleyi	12
FS1	Forest	300	T1	Homocropis_buckleyi	10
FS1	Forest	300	T2	Homocropis_buckleyi	8
FS1	Forest	300	T3	Homocropis_buckleyi	12
FS2	Forest	0	T1	Homocropis_buckleyi	6
FS2	Forest	0	T2	Homocropis_buckleyi	7

FS2	Forest	0	T3	Homocropis_buckleyi	11
FS2	Forest	150	T1	Homocropis_buckleyi	1
FS2	Forest	150	T2	Homocropis_buckleyi	2
FS2	Forest	150	T3	Homocropis_buckleyi	1
FS2	Forest	300	T1	Homocropis_buckleyi	9
FS2	Forest	300	T2	Homocropis_buckleyi	4
FS2	Forest	300	T3	Homocropis_buckleyi	5
PS2	Pinus	0	T1	Homocropis_buckleyi	1
PS2	Pinus	0	T3	Homocropis_buckleyi	4
PS2	Pinus	150	T1	Homocropis_buckleyi	3
PS2	Pinus	150	T2	Homocropis_buckleyi	2
PS2	Pinus	150	T3	Homocropis_buckleyi	1
PS2	Pinus	300	T1	Homocropis_buckleyi	1
PS2	Pinus	300	T2	Homocropis_buckleyi	1
PS2	Pinus	300	T3	Homocropis_buckleyi	3
PS1	Pinus	150	T3	Onthophagus_curvicornis	1
PS1	Pinus	300	T2	Dichotomius_cotopaxi	1
PS1	Pinus	300	T2	Dichotomius_cotopaxi	1
ES2	Eucalyptus	0	T3	Dichotomius_cotopaxi	1
ES2	Eucalyptus	150	T2	Dichotomius_cotopaxi	1
ES2	Eucalyptus	300	T1	Dichotomius_cotopaxi	1
ES2	Eucalyptus	300	T2	Dichotomius_cotopaxi	7
ES1	Eucalyptus	150	T3	Dichotomius_cotopaxi	1
ES1	Eucalyptus	150	T3	Dichotomius_cotopaxi	1
ES1	Eucalyptus	150	T3	Dichotomius_cotopaxi	5

Table S2. Measurements of morphological traits by species across different land-use types

Species	Ind	Land_Use	Plot_ID	Trait	Value
Dichotomius_cotopaxi	1	Pinus	PL2	HL	0.60
Dichotomius_cotopaxi	1	Pinus	PL2	HW	0.70
Dichotomius_cotopaxi	1	Pinus	PL2	PL	0.75
Dichotomius_cotopaxi	1	Pinus	PL2	PW	1.20
Dichotomius_cotopaxi	1	Pinus	PL2	PH	0.7
Dichotomius_cotopaxi	1	Pinus	PL2	EL	1.00
Dichotomius_cotopaxi	1	Pinus	PL2	pTL	0.45
Dichotomius_cotopaxi	1	Pinus	PL2	pTW	0.19
Dichotomius_cotopaxi	1	Pinus	PL2	mTL	0.44
Dichotomius_cotopaxi	1	Pinus	PL2	Biomass	0.00286
Dichotomius_cotopaxi	2	Pinus	PL2	HL	0.46
Dichotomius_cotopaxi	2	Pinus	PL2	HW	0.70
Dichotomius_cotopaxi	2	Pinus	PL2	PL	0.72
Dichotomius_cotopaxi	2	Pinus	PL2	PW	1.20
Dichotomius_cotopaxi	2	Pinus	PL2	PH	0.75
Dichotomius_cotopaxi	2	Pinus	PL2	EL	0.95
Dichotomius_cotopaxi	2	Pinus	PL2	pTL	0.45
Dichotomius_cotopaxi	2	Pinus	PL2	pTW	0.20
Dichotomius_cotopaxi	2	Pinus	PL2	mTL	0.40
Dichotomius_cotopaxi	2	Pinus	PL2	Biomass	0.00221
Dichotomius_cotopaxi	3	Pinus	PL2	HL	0.50
Dichotomius_cotopaxi	3	Pinus	PL2	HW	0.65
Dichotomius_cotopaxi	3	Pinus	PL2	PL	0.65
Dichotomius_cotopaxi	3	Pinus	PL2	PW	1.10
Dichotomius_cotopaxi	3	Pinus	PL2	PH	0.62
Dichotomius_cotopaxi	3	Pinus	PL2	EL	1.05
Dichotomius_cotopaxi	3	Pinus	PL2	pTL	0.35
Dichotomius_cotopaxi	3	Pinus	PL2	pTW	0.18
Dichotomius_cotopaxi	3	Pinus	PL2	mTL	0.43
Dichotomius_cotopaxi	3	Pinus	PL2	Biomass	0.0024
Dichotomius_cotopaxi	4	Pinus	PL2	HL	0.53
Dichotomius_cotopaxi	4	Pinus	PL2	HW	0.65
Dichotomius_cotopaxi	4	Pinus	PL2	PL	0.66
Dichotomius_cotopaxi	4	Pinus	PL2	PW	1.17
Dichotomius_cotopaxi	4	Pinus	PL2	PH	0.60
Dichotomius_cotopaxi	4	Pinus	PL2	EL	0.95
Dichotomius_cotopaxi	4	Pinus	PL2	pTL	0.35
Dichotomius_cotopaxi	4	Pinus	PL2	pTW	0.18
Dichotomius_cotopaxi	4	Pinus	PL2	mTL	0.41
Dichotomius_cotopaxi	4	Pinus	PL2	Biomass	0.00223

Dichotomius_cotopaxi	1	Pinus	PL1	HL	0.52
Dichotomius_cotopaxi	1	Pinus	PL1	HW	0.79
Dichotomius_cotopaxi	1	Pinus	PL1	PL	0.90
Dichotomius_cotopaxi	1	Pinus	PL1	PW	1.45
Dichotomius_cotopaxi	1	Pinus	PL1	PH	0.75
Dichotomius_cotopaxi	1	Pinus	PL1	EL	1.20
Dichotomius_cotopaxi	1	Pinus	PL1	pTL	0.48
Dichotomius_cotopaxi	1	Pinus	PL1	pTW	0.22
Dichotomius_cotopaxi	1	Pinus	PL1	mTL	0.45
Dichotomius_cotopaxi	1	Pinus	PL1	Biomass	0.0038
Dichotomius_cotopaxi	1	Forest	FL1	HL	0.50
Dichotomius_cotopaxi	1	Forest	FL1	HW	0.65
Dichotomius_cotopaxi	1	Forest	FL1	PL	0.68
Dichotomius_cotopaxi	1	Forest	FL1	PW	0.12
Dichotomius_cotopaxi	1	Forest	FL1	PH	0.65
Dichotomius_cotopaxi	1	Forest	FL1	EL	0.95
Dichotomius_cotopaxi	1	Forest	FL1	pTL	0.40
Dichotomius_cotopaxi	1	Forest	FL1	pTW	0.18
Dichotomius_cotopaxi	1	Forest	FL1	mTL	0.43
Dichotomius_cotopaxi	1	Forest	FL1	Biomass	0.00221
Dichotomius_cotopaxi	2	Forest	FL1	HL	0.60
Dichotomius_cotopaxi	2	Forest	FL1	HW	0.72
Dichotomius_cotopaxi	2	Forest	FL1	PL	0.73
Dichotomius_cotopaxi	2	Forest	FL1	PW	1.25
Dichotomius_cotopaxi	2	Forest	FL1	PH	0.66
Dichotomius_cotopaxi	2	Forest	FL1	EL	1.10
Dichotomius_cotopaxi	2	Forest	FL1	pTL	0.40
Dichotomius_cotopaxi	2	Forest	FL1	pTW	0.18
Dichotomius_cotopaxi	2	Forest	FL1	mTL	0.45
Dichotomius_cotopaxi	2	Forest	FL1	Biomass	0.00312
Dichotomius_cotopaxi	1	Forest	FL2	HL	0.43
Dichotomius_cotopaxi	1	Forest	FL2	HW	0.72
Dichotomius_cotopaxi	1	Forest	FL2	PL	0.66
Dichotomius_cotopaxi	1	Forest	FL2	PW	1.10
Dichotomius_cotopaxi	1	Forest	FL2	PH	0.65
Dichotomius_cotopaxi	1	Forest	FL2	EL	0.97
Dichotomius_cotopaxi	1	Forest	FL2	pTL	0.42
Dichotomius_cotopaxi	1	Forest	FL2	pTW	0.20
Dichotomius_cotopaxi	1	Forest	FL2	mTL	0.47
Dichotomius_cotopaxi	1	Forest	FL2	Biomass	0.00202
Dichotomius_cotopaxi	2	Forest	FL2	HL	0.42
Dichotomius_cotopaxi	2	Forest	FL2	HW	0.80
Dichotomius_cotopaxi	2	Forest	FL2	PL	0.85

Dichotomius_cotopaxi	2	Forest	FL2	PW	1.50
Dichotomius_cotopaxi	2	Forest	FL2	PH	0.80
Dichotomius_cotopaxi	2	Forest	FL2	EL	0.45
Dichotomius_cotopaxi	2	Forest	FL2	pTL	0.52
Dichotomius_cotopaxi	2	Forest	FL2	pTW	0.25
Dichotomius_cotopaxi	2	Forest	FL2	mTL	0.62
Dichotomius_cotopaxi	2	Forest	FL2	Biomass	0.00126
Dichotomius_cotopaxi	1	Eucalyptus	EL2	HL	0.50
Dichotomius_cotopaxi	1	Eucalyptus	EL2	HW	0.68
Dichotomius_cotopaxi	1	Eucalyptus	EL2	PL	0.75
Dichotomius_cotopaxi	1	Eucalyptus	EL2	PW	1.20
Dichotomius_cotopaxi	1	Eucalyptus	EL2	PH	0.60
Dichotomius_cotopaxi	1	Eucalyptus	EL2	EL	1.00
Dichotomius_cotopaxi	1	Eucalyptus	EL2	pTL	0.42
Dichotomius_cotopaxi	1	Eucalyptus	EL2	pTW	0.20
Dichotomius_cotopaxi	1	Eucalyptus	EL2	mTL	0.45
Dichotomius_cotopaxi	1	Eucalyptus	EL2	Biomass	0.00221
Onthophagus_curvicornis	1	Forest	FL2	HL	0.17
Onthophagus_curvicornis	1	Forest	FL2	HW	0.24
Onthophagus_curvicornis	1	Forest	FL2	PL	0.3
Onthophagus_curvicornis	1	Forest	FL2	PW	0.43
Onthophagus_curvicornis	1	Forest	FL2	PH	0.24
Onthophagus_curvicornis	1	Forest	FL2	EL	0.45
Onthophagus_curvicornis	1	Forest	FL2	pTL	0.19
Onthophagus_curvicornis	1	Forest	FL2	pTW	0.05
Onthophagus_curvicornis	1	Forest	FL2	mTL	0.16
Onthophagus_curvicornis	1	Forest	FL2	Biomass	0.00024
Onthophagus_curvicornis	2	Forest	FL2	HL	0.2
Onthophagus_curvicornis	2	Forest	FL2	HW	0.28
Onthophagus_curvicornis	2	Forest	FL2	PL	0.34
Onthophagus_curvicornis	2	Forest	FL2	PW	0.48
Onthophagus_curvicornis	2	Forest	FL2	PH	0.26
Onthophagus_curvicornis	2	Forest	FL2	EL	0.42
Onthophagus_curvicornis	2	Forest	FL2	pTL	0.15
Onthophagus_curvicornis	2	Forest	FL2	pTW	0.04
Onthophagus_curvicornis	2	Forest	FL2	mTL	0.17
Onthophagus_curvicornis	2	Forest	FL2	Biomass	0.00027
Onthophagus_curvicornis	3	Forest	FL2	HL	0.22
Onthophagus_curvicornis	3	Forest	FL2	HW	0.27
Onthophagus_curvicornis	3	Forest	FL2	PL	0.35
Onthophagus_curvicornis	3	Forest	FL2	PW	0.5
Onthophagus_curvicornis	3	Forest	FL2	PH	0.28
Onthophagus_curvicornis	3	Forest	FL2	EL	0.38

Onthophagus_curvicornis	3	Forest	FL2	pTL	0.18
Onthophagus_curvicornis	3	Forest	FL2	pTW	0.07
Onthophagus_curvicornis	3	Forest	FL2	mTL	0.15
Onthophagus_curvicornis	3	Forest	FL2	Biomass	0.00026
Onthophagus_curvicornis	4	Forest	FL2	HL	0.18
Onthophagus_curvicornis	4	Forest	FL2	HW	0.23
Onthophagus_curvicornis	4	Forest	FL2	PL	0.29
Onthophagus_curvicornis	4	Forest	FL2	PW	0.43
Onthophagus_curvicornis	4	Forest	FL2	PH	0.25
Onthophagus_curvicornis	4	Forest	FL2	EL	0.35
Onthophagus_curvicornis	4	Forest	FL2	pTL	0.14
Onthophagus_curvicornis	4	Forest	FL2	pTW	0.05
Onthophagus_curvicornis	4	Forest	FL2	mTL	0.15
Onthophagus_curvicornis	4	Forest	FL2	Biomass	0.00018
Onthophagus_curvicornis	5	Forest	FL2	HL	0.27
Onthophagus_curvicornis	5	Forest	FL2	HW	0.3
Onthophagus_curvicornis	5	Forest	FL2	PL	0.35
Onthophagus_curvicornis	5	Forest	FL2	PW	0.5
Onthophagus_curvicornis	5	Forest	FL2	PH	0.2
Onthophagus_curvicornis	5	Forest	FL2	EL	0.4
Onthophagus_curvicornis	5	Forest	FL2	pTL	0.2
Onthophagus_curvicornis	5	Forest	FL2	pTW	0.06
Onthophagus_curvicornis	5	Forest	FL2	mTL	0.14
Onthophagus_curvicornis	5	Forest	FL2	Biomass	0.00032
Onthophagus_curvicornis	6	Forest	FL2	HL	0.2
Onthophagus_curvicornis	6	Forest	FL2	HW	0.22
Onthophagus_curvicornis	6	Forest	FL2	PL	0.3
Onthophagus_curvicornis	6	Forest	FL2	PW	0.45
Onthophagus_curvicornis	6	Forest	FL2	PH	0.27
Onthophagus_curvicornis	6	Forest	FL2	EL	0.4
Onthophagus_curvicornis	6	Forest	FL2	pTL	0.2
Onthophagus_curvicornis	6	Forest	FL2	pTW	0.05
Onthophagus_curvicornis	6	Forest	FL2	mTL	0.17
Onthophagus_curvicornis	6	Forest	FL2	Biomass	0.00023
Onthophagus_curvicornis	7	Forest	FL2	HL	0.22
Onthophagus_curvicornis	7	Forest	FL2	HW	0.26
Onthophagus_curvicornis	7	Forest	FL2	PL	0.3
Onthophagus_curvicornis	7	Forest	FL2	PW	0.47
Onthophagus_curvicornis	7	Forest	FL2	PH	0.23
Onthophagus_curvicornis	7	Forest	FL2	EL	0.45
Onthophagus_curvicornis	7	Forest	FL2	pTL	0.15
Onthophagus_curvicornis	7	Forest	FL2	pTW	0.04
Onthophagus_curvicornis	7	Forest	FL2	mTL	0.17

Onthophagus_curvicornis	7	Forest	FL2	Biomass	0.00028
Onthophagus_curvicornis	8	Forest	FL2	HL	0.25
Onthophagus_curvicornis	8	Forest	FL2	HW	0.27
Onthophagus_curvicornis	8	Forest	FL2	PL	0.3
Onthophagus_curvicornis	8	Forest	FL2	PW	0.46
Onthophagus_curvicornis	8	Forest	FL2	PH	0.25
Onthophagus_curvicornis	8	Forest	FL2	EL	0.45
Onthophagus_curvicornis	8	Forest	FL2	pTL	0.21
Onthophagus_curvicornis	8	Forest	FL2	pTW	0.08
Onthophagus_curvicornis	8	Forest	FL2	mTL	0.15
Onthophagus_curvicornis	8	Forest	FL2	Biomass	0.000305
Onthophagus_curvicornis	9	Forest	FL2	HL	0.15
Onthophagus_curvicornis	9	Forest	FL2	HW	0.25
Onthophagus_curvicornis	9	Forest	FL2	PL	0.33
Onthophagus_curvicornis	9	Forest	FL2	PW	0.45
Onthophagus_curvicornis	9	Forest	FL2	PH	0.2
Onthophagus_curvicornis	9	Forest	FL2	EL	0.37
Onthophagus_curvicornis	9	Forest	FL2	pTL	0.22
Onthophagus_curvicornis	9	Forest	FL2	pTW	0.06
Onthophagus_curvicornis	9	Forest	FL2	mTL	0.18
Onthophagus_curvicornis	9	Forest	FL2	Biomass	0.00019
Onthophagus_curvicornis	10	Forest	FL2	HL	0.22
Onthophagus_curvicornis	10	Forest	FL2	HW	0.28
Onthophagus_curvicornis	10	Forest	FL2	PL	0.36
Onthophagus_curvicornis	10	Forest	FL2	PW	0.51
Onthophagus_curvicornis	10	Forest	FL2	PH	0.25
Onthophagus_curvicornis	10	Forest	FL2	EL	0.44
Onthophagus_curvicornis	10	Forest	FL2	pTL	0.22
Onthophagus_curvicornis	10	Forest	FL2	pTW	0.07
Onthophagus_curvicornis	10	Forest	FL2	mTL	0.17
Onthophagus_curvicornis	10	Forest	FL2	Biomass	0.00032
Onthophagus_curvicornis	1	Pinus	PL1	HL	0.2
Onthophagus_curvicornis	1	Pinus	PL1	HW	0.27
Onthophagus_curvicornis	1	Pinus	PL1	PL	0.39
Onthophagus_curvicornis	1	Pinus	PL1	PW	0.5
Onthophagus_curvicornis	1	Pinus	PL1	PH	0.35
Onthophagus_curvicornis	1	Pinus	PL1	EL	0.4
Onthophagus_curvicornis	1	Pinus	PL1	pTL	0.23
Onthophagus_curvicornis	1	Pinus	PL1	pTW	0.07
Onthophagus_curvicornis	1	Pinus	PL1	mTL	0.2
Onthophagus_curvicornis	1	Pinus	PL1	Biomass	0.00029
Onthophagus_curvicornis	2	Pinus	PL1	HL	0.25
Onthophagus_curvicornis	2	Pinus	PL1	HW	0.29

Onthophagus_curvicornis	2	Pinus	PL1	PL	0.45
Onthophagus_curvicornis	2	Pinus	PL1	PW	0.55
Onthophagus_curvicornis	2	Pinus	PL1	PH	0.38
Onthophagus_curvicornis	2	Pinus	PL1	EL	0.46
Onthophagus_curvicornis	2	Pinus	PL1	pTL	0.3
Onthophagus_curvicornis	2	Pinus	PL1	pTW	0.07
Onthophagus_curvicornis	2	Pinus	PL1	mTL	0.2
Onthophagus_curvicornis	2	Pinus	PL1	Biomass	0.00044
Onthophagus_curvicornis	3	Pinus	PL1	HL	0.24
Onthophagus_curvicornis	3	Pinus	PL1	HW	0.3
Onthophagus_curvicornis	3	Pinus	PL1	PL	0.42
Onthophagus_curvicornis	3	Pinus	PL1	PW	0.58
Onthophagus_curvicornis	3	Pinus	PL1	PH	0.41
Onthophagus_curvicornis	3	Pinus	PL1	EL	0.4
Onthophagus_curvicornis	3	Pinus	PL1	pTL	0.24
Onthophagus_curvicornis	3	Pinus	PL1	pTW	0.08
Onthophagus_curvicornis	3	Pinus	PL1	mTL	0.18
Onthophagus_curvicornis	3	Pinus	PL1	Biomass	0.00035
Onthophagus_curvicornis	4	Pinus	PL1	HL	0.25
Onthophagus_curvicornis	4	Pinus	PL1	HW	0.28
Onthophagus_curvicornis	4	Pinus	PL1	PL	0.35
Onthophagus_curvicornis	4	Pinus	PL1	PW	0.5
Onthophagus_curvicornis	4	Pinus	PL1	PH	0.3
Onthophagus_curvicornis	4	Pinus	PL1	EL	0.38
Onthophagus_curvicornis	4	Pinus	PL1	pTL	0.23
Onthophagus_curvicornis	4	Pinus	PL1	pTW	0.05
Onthophagus_curvicornis	4	Pinus	PL1	mTL	0.16
Onthophagus_curvicornis	4	Pinus	PL1	Biomass	0.00028
Onthophagus_curvicornis	5	Pinus	PL1	HL	0.22
Onthophagus_curvicornis	5	Pinus	PL1	HW	0.26
Onthophagus_curvicornis	5	Pinus	PL1	PL	0.32
Onthophagus_curvicornis	5	Pinus	PL1	PW	0.45
Onthophagus_curvicornis	5	Pinus	PL1	PH	0.25
Onthophagus_curvicornis	5	Pinus	PL1	EL	0.32
Onthophagus_curvicornis	5	Pinus	PL1	pTL	0.16
Onthophagus_curvicornis	5	Pinus	PL1	pTW	0.04
Onthophagus_curvicornis	5	Pinus	PL1	mTL	0.15
Onthophagus_curvicornis	5	Pinus	PL1	Biomass	0.0002
Onthophagus_curvicornis	6	Pinus	PL1	HL	0.22
Onthophagus_curvicornis	6	Pinus	PL1	HW	0.28
Onthophagus_curvicornis	6	Pinus	PL1	PL	0.3
Onthophagus_curvicornis	6	Pinus	PL1	PW	0.27
Onthophagus_curvicornis	6	Pinus	PL1	PH	0.33

Onthophagus_curvicornis	6	Pinus	PL1	EL	0.35
Onthophagus_curvicornis	6	Pinus	PL1	pTL	0.17
Onthophagus_curvicornis	6	Pinus	PL1	pTW	0.07
Onthophagus_curvicornis	6	Pinus	PL1	mTL	0.13
Onthophagus_curvicornis	6	Pinus	PL1	Biomass	0.00021
Onthophagus_curvicornis	7	Pinus	PL1	HL	0.29
Onthophagus_curvicornis	7	Pinus	PL1	HW	0.31
Onthophagus_curvicornis	7	Pinus	PL1	PL	0.4
Onthophagus_curvicornis	7	Pinus	PL1	PW	0.57
Onthophagus_curvicornis	7	Pinus	PL1	PH	0.33
Onthophagus_curvicornis	7	Pinus	PL1	EL	0.6
Onthophagus_curvicornis	7	Pinus	PL1	pTL	0.22
Onthophagus_curvicornis	7	Pinus	PL1	pTW	0.08
Onthophagus_curvicornis	7	Pinus	PL1	mTL	0.2
Onthophagus_curvicornis	7	Pinus	PL1	Biomass	0.00059
Onthophagus_curvicornis	8	Pinus	PL1	HL	0.21
Onthophagus_curvicornis	8	Pinus	PL1	HW	0.35
Onthophagus_curvicornis	8	Pinus	PL1	PL	0.52
Onthophagus_curvicornis	8	Pinus	PL1	PW	0.62
Onthophagus_curvicornis	8	Pinus	PL1	PH	0.3
Onthophagus_curvicornis	8	Pinus	PL1	EL	0.42
Onthophagus_curvicornis	8	Pinus	PL1	pTL	0.34
Onthophagus_curvicornis	8	Pinus	PL1	pTW	0.1
Onthophagus_curvicornis	8	Pinus	PL1	mTL	0.23
Onthophagus_curvicornis	8	Pinus	PL1	Biomass	0.00043
Onthophagus_curvicornis	9	Pinus	PL1	HL	0.22
Onthophagus_curvicornis	9	Pinus	PL1	HW	0.31
Onthophagus_curvicornis	9	Pinus	PL1	PL	0.4
Onthophagus_curvicornis	9	Pinus	PL1	PW	0.57
Onthophagus_curvicornis	9	Pinus	PL1	PH	0.25
Onthophagus_curvicornis	9	Pinus	PL1	EL	0.45
Onthophagus_curvicornis	9	Pinus	PL1	pTL	0.18
Onthophagus_curvicornis	9	Pinus	PL1	pTW	0.05
Onthophagus_curvicornis	9	Pinus	PL1	mTL	0.16
Onthophagus_curvicornis	9	Pinus	PL1	Biomass	0.00036
Onthophagus_curvicornis	10	Pinus	PL1	HL	0.25
Onthophagus_curvicornis	10	Pinus	PL1	HW	0.3
Onthophagus_curvicornis	10	Pinus	PL1	PL	0.39
Onthophagus_curvicornis	10	Pinus	PL1	PW	0.55
Onthophagus_curvicornis	10	Pinus	PL1	PH	0.35
Onthophagus_curvicornis	10	Pinus	PL1	EL	0.46
Onthophagus_curvicornis	10	Pinus	PL1	pTL	0.2
Onthophagus_curvicornis	10	Pinus	PL1	pTW	0.06

Onthophagus_curvicornis	10	Pinus	PL1	mTL	0.18
Onthophagus_curvicornis	10	Pinus	PL1	Biomass	0.00039
Onthophagus_curvicornis	1	Eucalyptus	EL2	HL	0.18
Onthophagus_curvicornis	1	Eucalyptus	EL2	HW	0.23
Onthophagus_curvicornis	1	Eucalyptus	EL2	PL	0.27
Onthophagus_curvicornis	1	Eucalyptus	EL2	PW	0.41
Onthophagus_curvicornis	1	Eucalyptus	EL2	PH	0.21
Onthophagus_curvicornis	1	Eucalyptus	EL2	EL	0.36
Onthophagus_curvicornis	1	Eucalyptus	EL2	pTL	0.14
Onthophagus_curvicornis	1	Eucalyptus	EL2	pTW	0.02
Onthophagus_curvicornis	1	Eucalyptus	EL2	mTL	0.16
Onthophagus_curvicornis	1	Eucalyptus	EL2	Biomass	0.00017
Onthophagus_curvicornis	2	Eucalyptus	EL2	HL	0.12
Onthophagus_curvicornis	2	Eucalyptus	EL2	HW	0.2
Onthophagus_curvicornis	2	Eucalyptus	EL2	PL	0.22
Onthophagus_curvicornis	2	Eucalyptus	EL2	PW	0.35
Onthophagus_curvicornis	2	Eucalyptus	EL2	PH	0.15
Onthophagus_curvicornis	2	Eucalyptus	EL2	EL	0.38
Onthophagus_curvicornis	2	Eucalyptus	EL2	pTL	0.11
Onthophagus_curvicornis	2	Eucalyptus	EL2	pTW	0.05
Onthophagus_curvicornis	2	Eucalyptus	EL2	mTL	0.16
Onthophagus_curvicornis	2	Eucalyptus	EL2	Biomass	0.00012
Onthophagus_curvicornis	3	Eucalyptus	EL2	HL	0.12
Onthophagus_curvicornis	3	Eucalyptus	EL2	HW	0.19
Onthophagus_curvicornis	3	Eucalyptus	EL2	PL	0.25
Onthophagus_curvicornis	3	Eucalyptus	EL2	PW	0.35
Onthophagus_curvicornis	3	Eucalyptus	EL2	PH	0.16
Onthophagus_curvicornis	3	Eucalyptus	EL2	EL	0.38
Onthophagus_curvicornis	3	Eucalyptus	EL2	pTL	0.11
Onthophagus_curvicornis	3	Eucalyptus	EL2	pTW	0.06
Onthophagus_curvicornis	3	Eucalyptus	EL2	mTL	0.15
Onthophagus_curvicornis	3	Eucalyptus	EL2	Biomass	0.00014
Onthophagus_curvicornis	4	Eucalyptus	EL2	HL	0.2
Onthophagus_curvicornis	4	Eucalyptus	EL2	HW	0.22
Onthophagus_curvicornis	4	Eucalyptus	EL2	PL	0.4
Onthophagus_curvicornis	4	Eucalyptus	EL2	PW	0.4
Onthophagus_curvicornis	4	Eucalyptus	EL2	PH	0.21
Onthophagus_curvicornis	4	Eucalyptus	EL2	EL	0.39
Onthophagus_curvicornis	4	Eucalyptus	EL2	pTL	0.16
Onthophagus_curvicornis	4	Eucalyptus	EL2	pTW	0.04
Onthophagus_curvicornis	4	Eucalyptus	EL2	mTL	0.13
Onthophagus_curvicornis	4	Eucalyptus	EL2	Biomass	0.00029
Onthophagus_curvicornis	5	Eucalyptus	EL2	HL	0.26

Onthophagus_curvicornis	5	Eucalyptus	EL2	HW	0.3
Onthophagus_curvicornis	5	Eucalyptus	EL2	PL	0.38
Onthophagus_curvicornis	5	Eucalyptus	EL2	PW	0.58
Onthophagus_curvicornis	5	Eucalyptus	EL2	PH	0.28
Onthophagus_curvicornis	5	Eucalyptus	EL2	EL	0.47
Onthophagus_curvicornis	5	Eucalyptus	EL2	pTL	0.24
Onthophagus_curvicornis	5	Eucalyptus	EL2	pTW	0.06
Onthophagus_curvicornis	5	Eucalyptus	EL2	mTL	0.2
Onthophagus_curvicornis	5	Eucalyptus	EL2	Biomass	0.0004
Onthophagus_curvicornis	6	Eucalyptus	EL2	HL	0.25
Onthophagus_curvicornis	6	Eucalyptus	EL2	HW	0.3
Onthophagus_curvicornis	6	Eucalyptus	EL2	PL	0.41
Onthophagus_curvicornis	6	Eucalyptus	EL2	PW	0.55
Onthophagus_curvicornis	6	Eucalyptus	EL2	PH	0.3
Onthophagus_curvicornis	6	Eucalyptus	EL2	EL	0.43
Onthophagus_curvicornis	6	Eucalyptus	EL2	pTL	0.24
Onthophagus_curvicornis	6	Eucalyptus	EL2	pTW	0.06
Onthophagus_curvicornis	6	Eucalyptus	EL2	mTL	0.19
Onthophagus_curvicornis	6	Eucalyptus	EL2	Biomass	0.00038
Onthophagus_curvicornis	7	Eucalyptus	EL2	HL	0.24
Onthophagus_curvicornis	7	Eucalyptus	EL2	HW	0.3
Onthophagus_curvicornis	7	Eucalyptus	EL2	PL	0.35
Onthophagus_curvicornis	7	Eucalyptus	EL2	PW	0.5
Onthophagus_curvicornis	7	Eucalyptus	EL2	PH	0.32
Onthophagus_curvicornis	7	Eucalyptus	EL2	EL	0.4
Onthophagus_curvicornis	7	Eucalyptus	EL2	pTL	0.16
Onthophagus_curvicornis	7	Eucalyptus	EL2	pTW	0.07
Onthophagus_curvicornis	7	Eucalyptus	EL2	mTL	0.18
Onthophagus_curvicornis	7	Eucalyptus	EL2	Biomass	0.00029
Onthophagus_curvicornis	8	Eucalyptus	EL2	HL	0.25
Onthophagus_curvicornis	8	Eucalyptus	EL2	HW	0.27
Onthophagus_curvicornis	8	Eucalyptus	EL2	PL	0.37
Onthophagus_curvicornis	8	Eucalyptus	EL2	PW	0.5
Onthophagus_curvicornis	8	Eucalyptus	EL2	PH	0.3
Onthophagus_curvicornis	8	Eucalyptus	EL2	EL	0.4
Onthophagus_curvicornis	8	Eucalyptus	EL2	pTL	0.23
Onthophagus_curvicornis	8	Eucalyptus	EL2	pTW	0.09
Onthophagus_curvicornis	8	Eucalyptus	EL2	mTL	0.18
Onthophagus_curvicornis	8	Eucalyptus	EL2	Biomass	0.00038
Onthophagus_curvicornis	9	Eucalyptus	EL2	HL	0.23
Onthophagus_curvicornis	9	Eucalyptus	EL2	HW	0.29
Onthophagus_curvicornis	9	Eucalyptus	EL2	PL	0.37
Onthophagus_curvicornis	9	Eucalyptus	EL2	PW	0.5

Onthophagus_curvicornis	9	Eucalyptus	EL2	PH	0.28
Onthophagus_curvicornis	9	Eucalyptus	EL2	EL	0.39
Onthophagus_curvicornis	9	Eucalyptus	EL2	pTL	0.16
Onthophagus_curvicornis	9	Eucalyptus	EL2	pTW	0.04
Onthophagus_curvicornis	9	Eucalyptus	EL2	mTL	0.14
Onthophagus_curvicornis	9	Eucalyptus	EL2	Biomass	0.00029
Onthophagus_curvicornis	10	Eucalyptus	EL2	HL	0.29
Onthophagus_curvicornis	10	Eucalyptus	EL2	HW	0.31
Onthophagus_curvicornis	10	Eucalyptus	EL2	PL	0.4
Onthophagus_curvicornis	10	Eucalyptus	EL2	PW	0.59
Onthophagus_curvicornis	10	Eucalyptus	EL2	PH	0.3
Onthophagus_curvicornis	10	Eucalyptus	EL2	EL	0.35
Onthophagus_curvicornis	10	Eucalyptus	EL2	pTL	0.29
Onthophagus_curvicornis	10	Eucalyptus	EL2	pTW	0.11
Onthophagus_curvicornis	10	Eucalyptus	EL2	mTL	0.21
Onthophagus_curvicornis	10	Eucalyptus	EL2	Biomass	0.00033
Onthophagus_curvicornis	1	Pinus	PL2	HL	0.25
Onthophagus_curvicornis	1	Pinus	PL2	HW	0.31
Onthophagus_curvicornis	1	Pinus	PL2	PL	0.4
Onthophagus_curvicornis	1	Pinus	PL2	PW	0.55
Onthophagus_curvicornis	1	Pinus	PL2	PH	0.28
Onthophagus_curvicornis	1	Pinus	PL2	EL	0.42
Onthophagus_curvicornis	1	Pinus	PL2	pTL	0.19
Onthophagus_curvicornis	1	Pinus	PL2	pTW	0.05
Onthophagus_curvicornis	1	Pinus	PL2	mTL	0.2
Onthophagus_curvicornis	1	Pinus	PL2	Biomass	0.00036
Onthophagus_curvicornis	2	Pinus	PL2	HL	0.25
Onthophagus_curvicornis	2	Pinus	PL2	HW	0.29
Onthophagus_curvicornis	2	Pinus	PL2	PL	0.46
Onthophagus_curvicornis	2	Pinus	PL2	PW	0.53
Onthophagus_curvicornis	2	Pinus	PL2	PH	0.32
Onthophagus_curvicornis	2	Pinus	PL2	EL	0.45
Onthophagus_curvicornis	2	Pinus	PL2	pTL	0.15
Onthophagus_curvicornis	2	Pinus	PL2	pTW	0.05
Onthophagus_curvicornis	2	Pinus	PL2	mTL	0.18
Onthophagus_curvicornis	2	Pinus	PL2	Biomass	0.00044
Onthophagus_curvicornis	3	Pinus	PL2	HL	0.23
Onthophagus_curvicornis	3	Pinus	PL2	HW	0.27
Onthophagus_curvicornis	3	Pinus	PL2	PL	0.35
Onthophagus_curvicornis	3	Pinus	PL2	PW	0.5
Onthophagus_curvicornis	3	Pinus	PL2	PH	0.28
Onthophagus_curvicornis	3	Pinus	PL2	EL	0.36
Onthophagus_curvicornis	3	Pinus	PL2	pTL	0.19

Onthophagus_curvicornis	3	Pinus	PL2	pTW	0.08
Onthophagus_curvicornis	3	Pinus	PL2	mTL	0.2
Onthophagus_curvicornis	3	Pinus	PL2	Biomass	0.00025
Onthophagus_curvicornis	4	Pinus	PL2	HL	0.23
Onthophagus_curvicornis	4	Pinus	PL2	HW	0.3
Onthophagus_curvicornis	4	Pinus	PL2	PL	0.35
Onthophagus_curvicornis	4	Pinus	PL2	PW	0.53
Onthophagus_curvicornis	4	Pinus	PL2	PH	0.28
Onthophagus_curvicornis	4	Pinus	PL2	EL	0.43
Onthophagus_curvicornis	4	Pinus	PL2	pTL	0.18
Onthophagus_curvicornis	4	Pinus	PL2	pTW	0.06
Onthophagus_curvicornis	4	Pinus	PL2	mTL	0.19
Onthophagus_curvicornis	4	Pinus	PL2	Biomass	0.00031
Onthophagus_curvicornis	5	Pinus	PL2	HL	0.22
Onthophagus_curvicornis	5	Pinus	PL2	HW	0.33
Onthophagus_curvicornis	5	Pinus	PL2	PL	0.23
Onthophagus_curvicornis	5	Pinus	PL2	PW	0.58
Onthophagus_curvicornis	5	Pinus	PL2	PH	0.3
Onthophagus_curvicornis	5	Pinus	PL2	EL	0.39
Onthophagus_curvicornis	5	Pinus	PL2	pTL	0.3
Onthophagus_curvicornis	5	Pinus	PL2	pTW	0.09
Onthophagus_curvicornis	5	Pinus	PL2	mTL	0.22
Onthophagus_curvicornis	5	Pinus	PL2	Biomass	0.00019
Onthophagus_curvicornis	6	Pinus	PL2	HL	0.21
Onthophagus_curvicornis	6	Pinus	PL2	HW	0.26
Onthophagus_curvicornis	6	Pinus	PL2	PL	0.46
Onthophagus_curvicornis	6	Pinus	PL2	PW	0.47
Onthophagus_curvicornis	6	Pinus	PL2	PH	0.27
Onthophagus_curvicornis	6	Pinus	PL2	EL	0.38
Onthophagus_curvicornis	6	Pinus	PL2	pTL	0.23
Onthophagus_curvicornis	6	Pinus	PL2	pTW	0.08
Onthophagus_curvicornis	6	Pinus	PL2	mTL	0.17
Onthophagus_curvicornis	6	Pinus	PL2	Biomass	0.00034
Onthophagus_curvicornis	7	Pinus	PL2	HL	0.25
Onthophagus_curvicornis	7	Pinus	PL2	HW	0.31
Onthophagus_curvicornis	7	Pinus	PL2	PL	0.39
Onthophagus_curvicornis	7	Pinus	PL2	PW	0.57
Onthophagus_curvicornis	7	Pinus	PL2	PH	0.28
Onthophagus_curvicornis	7	Pinus	PL2	EL	0.42
Onthophagus_curvicornis	7	Pinus	PL2	pTL	0.26
Onthophagus_curvicornis	7	Pinus	PL2	pTW	0.1
Onthophagus_curvicornis	7	Pinus	PL2	mTL	0.21
Onthophagus_curvicornis	7	Pinus	PL2	Biomass	0.00035

Onthophagus_curvicornis	8	Pinus	PL2	HL	0.26
Onthophagus_curvicornis	8	Pinus	PL2	HW	0.3
Onthophagus_curvicornis	8	Pinus	PL2	PL	0.4
Onthophagus_curvicornis	8	Pinus	PL2	PW	0.56
Onthophagus_curvicornis	8	Pinus	PL2	PH	0.28
Onthophagus_curvicornis	8	Pinus	PL2	EL	0.43
Onthophagus_curvicornis	8	Pinus	PL2	pTL	0.22
Onthophagus_curvicornis	8	Pinus	PL2	pTW	0.04
Onthophagus_curvicornis	8	Pinus	PL2	mTL	0.23
Onthophagus_curvicornis	8	Pinus	PL2	Biomass	0.00038
Onthophagus_curvicornis	9	Pinus	PL2	HL	0.23
Onthophagus_curvicornis	9	Pinus	PL2	HW	0.31
Onthophagus_curvicornis	9	Pinus	PL2	PL	0.4
Onthophagus_curvicornis	9	Pinus	PL2	PW	0.57
Onthophagus_curvicornis	9	Pinus	PL2	PH	0.25
Onthophagus_curvicornis	9	Pinus	PL2	EL	0.45
Onthophagus_curvicornis	9	Pinus	PL2	pTL	0.18
Onthophagus_curvicornis	9	Pinus	PL2	pTW	0.05
Onthophagus_curvicornis	9	Pinus	PL2	mTL	0.16
Onthophagus_curvicornis	9	Pinus	PL2	Biomass	0.00037
Onthophagus_curvicornis	10	Pinus	PL2	HL	0.23
Onthophagus_curvicornis	10	Pinus	PL2	HW	0.25
Onthophagus_curvicornis	10	Pinus	PL2	PL	0.34
Onthophagus_curvicornis	10	Pinus	PL2	PW	0.48
Onthophagus_curvicornis	10	Pinus	PL2	PH	0.22
Onthophagus_curvicornis	10	Pinus	PL2	EL	0.4
Onthophagus_curvicornis	10	Pinus	PL2	pTL	0.17
Onthophagus_curvicornis	10	Pinus	PL2	pTW	0.03
Onthophagus_curvicornis	10	Pinus	PL2	mTL	0.16
Onthophagus_curvicornis	10	Pinus	PL2	Biomass	0.00028
Onthophagus_curvicornis	1	Eucalyptus	EL1	HL	0.21
Onthophagus_curvicornis	1	Eucalyptus	EL1	HW	0.3
Onthophagus_curvicornis	1	Eucalyptus	EL1	PL	0.4
Onthophagus_curvicornis	1	Eucalyptus	EL1	PW	0.52
Onthophagus_curvicornis	1	Eucalyptus	EL1	PH	0.25
Onthophagus_curvicornis	1	Eucalyptus	EL1	EL	0.38
Onthophagus_curvicornis	1	Eucalyptus	EL1	pTL	0.2
Onthophagus_curvicornis	1	Eucalyptus	EL1	pTW	0.08
Onthophagus_curvicornis	1	Eucalyptus	EL1	mTL	0.16
Onthophagus_curvicornis	1	Eucalyptus	EL1	Biomass	0.00029
Onthophagus_curvicornis	2	Eucalyptus	EL1	HL	0.19
Onthophagus_curvicornis	2	Eucalyptus	EL1	HW	0.24
Onthophagus_curvicornis	2	Eucalyptus	EL1	PL	0.28

Onthophagus_curvicornis	2	Eucalyptus	EL1	PW	0.45
Onthophagus_curvicornis	2	Eucalyptus	EL1	PH	0.25
Onthophagus_curvicornis	2	Eucalyptus	EL1	EL	0.35
Onthophagus_curvicornis	2	Eucalyptus	EL1	pTL	0.16
Onthophagus_curvicornis	2	Eucalyptus	EL1	pTW	0.07
Onthophagus_curvicornis	2	Eucalyptus	EL1	mTL	0.15
Onthophagus_curvicornis	2	Eucalyptus	EL1	Biomass	0.00018
Onthophagus_curvicornis	3	Eucalyptus	EL1	HL	0.3
Onthophagus_curvicornis	3	Eucalyptus	EL1	HW	0.32
Onthophagus_curvicornis	3	Eucalyptus	EL1	PL	0.42
Onthophagus_curvicornis	3	Eucalyptus	EL1	PW	0.6
Onthophagus_curvicornis	3	Eucalyptus	EL1	PH	0.35
Onthophagus_curvicornis	3	Eucalyptus	EL1	EL	0.5
Onthophagus_curvicornis	3	Eucalyptus	EL1	pTL	0.22
Onthophagus_curvicornis	3	Eucalyptus	EL1	pTW	0.07
Onthophagus_curvicornis	3	Eucalyptus	EL1	mTL	0.18
Onthophagus_curvicornis	3	Eucalyptus	EL1	Biomass	0.00051
Onthophagus_curvicornis	4	Eucalyptus	EL1	HL	0.3
Onthophagus_curvicornis	4	Eucalyptus	EL1	HW	0.33
Onthophagus_curvicornis	4	Eucalyptus	EL1	PL	0.45
Onthophagus_curvicornis	4	Eucalyptus	EL1	PW	0.63
Onthophagus_curvicornis	4	Eucalyptus	EL1	PH	0.27
Onthophagus_curvicornis	4	Eucalyptus	EL1	EL	0.45
Onthophagus_curvicornis	4	Eucalyptus	EL1	pTL	0.22
Onthophagus_curvicornis	4	Eucalyptus	EL1	pTW	0.08
Onthophagus_curvicornis	4	Eucalyptus	EL1	mTL	0.2
Onthophagus_curvicornis	4	Eucalyptus	EL1	Biomass	0.00049
Onthophagus_curvicornis	5	Eucalyptus	EL1	HL	0.26
Onthophagus_curvicornis	5	Eucalyptus	EL1	HW	0.32
Onthophagus_curvicornis	5	Eucalyptus	EL1	PL	0.37
Onthophagus_curvicornis	5	Eucalyptus	EL1	PW	0.58
Onthophagus_curvicornis	5	Eucalyptus	EL1	PH	0.33
Onthophagus_curvicornis	5	Eucalyptus	EL1	EL	0.45
Onthophagus_curvicornis	5	Eucalyptus	EL1	pTL	0.22
Onthophagus_curvicornis	5	Eucalyptus	EL1	pTW	0.08
Onthophagus_curvicornis	5	Eucalyptus	EL1	mTL	0.2
Onthophagus_curvicornis	5	Eucalyptus	EL1	Biomass	0.00037
Onthophagus_curvicornis	6	Eucalyptus	EL1	HL	0.28
Onthophagus_curvicornis	6	Eucalyptus	EL1	HW	0.33
Onthophagus_curvicornis	6	Eucalyptus	EL1	PL	0.47
Onthophagus_curvicornis	6	Eucalyptus	EL1	PW	0.6
Onthophagus_curvicornis	6	Eucalyptus	EL1	PH	0.35
Onthophagus_curvicornis	6	Eucalyptus	EL1	EL	0.45

Onthophagus_curvicornis	6	Eucalyptus	EL1	pTL	0.32
Onthophagus_curvicornis	6	Eucalyptus	EL1	pTW	0.09
Onthophagus_curvicornis	6	Eucalyptus	EL1	mTL	0.18
Onthophagus_curvicornis	6	Eucalyptus	EL1	Biomass	0.00049
Onthophagus_curvicornis	7	Eucalyptus	EL1	HL	0.2
Onthophagus_curvicornis	7	Eucalyptus	EL1	HW	0.25
Onthophagus_curvicornis	7	Eucalyptus	EL1	PL	0.3
Onthophagus_curvicornis	7	Eucalyptus	EL1	PW	0.46
Onthophagus_curvicornis	7	Eucalyptus	EL1	PH	0.25
Onthophagus_curvicornis	7	Eucalyptus	EL1	EL	0.35
Onthophagus_curvicornis	7	Eucalyptus	EL1	pTL	0.18
Onthophagus_curvicornis	7	Eucalyptus	EL1	pTW	0.05
Onthophagus_curvicornis	7	Eucalyptus	EL1	mTL	0.16
Onthophagus_curvicornis	7	Eucalyptus	EL1	Biomass	0.00019
Onthophagus_curvicornis	8	Eucalyptus	EL1	HL	0.2
Onthophagus_curvicornis	8	Eucalyptus	EL1	HW	0.25
Onthophagus_curvicornis	8	Eucalyptus	EL1	PL	0.3
Onthophagus_curvicornis	8	Eucalyptus	EL1	PW	0.4
Onthophagus_curvicornis	8	Eucalyptus	EL1	PH	0.24
Onthophagus_curvicornis	8	Eucalyptus	EL1	EL	0.35
Onthophagus_curvicornis	8	Eucalyptus	EL1	pTL	0.17
Onthophagus_curvicornis	8	Eucalyptus	EL1	pTW	0.04
Onthophagus_curvicornis	8	Eucalyptus	EL1	mTL	0.13
Onthophagus_curvicornis	8	Eucalyptus	EL1	Biomass	0.00019
Onthophagus_curvicornis	9	Eucalyptus	EL1	HL	0.26
Onthophagus_curvicornis	9	Eucalyptus	EL1	HW	0.31
Onthophagus_curvicornis	9	Eucalyptus	EL1	PL	0.4
Onthophagus_curvicornis	9	Eucalyptus	EL1	PW	0.6
Onthophagus_curvicornis	9	Eucalyptus	EL1	PH	0.3
Onthophagus_curvicornis	9	Eucalyptus	EL1	EL	0.45
Onthophagus_curvicornis	9	Eucalyptus	EL1	pTL	0.25
Onthophagus_curvicornis	9	Eucalyptus	EL1	pTW	0.08
Onthophagus_curvicornis	9	Eucalyptus	EL1	mTL	0.23
Onthophagus_curvicornis	9	Eucalyptus	EL1	Biomass	0.00040
Onthophagus_curvicornis	10	Eucalyptus	EL1	HL	0.22
Onthophagus_curvicornis	10	Eucalyptus	EL1	HW	0.28
Onthophagus_curvicornis	10	Eucalyptus	EL1	PL	0.35
Onthophagus_curvicornis	10	Eucalyptus	EL1	PW	0.51
Onthophagus_curvicornis	10	Eucalyptus	EL1	PH	0.28
Onthophagus_curvicornis	10	Eucalyptus	EL1	EL	0.4
Onthophagus_curvicornis	10	Eucalyptus	EL1	pTL	0.25
Onthophagus_curvicornis	10	Eucalyptus	EL1	pTW	0.06
Onthophagus_curvicornis	10	Eucalyptus	EL1	mTL	0.2

Onthophagus_curvicornis	10	Eucalyptus	EL1	Biomass	0.00028
Cryptocanthon_paradoxus	1	Forest	FL1	HL	0.12
Cryptocanthon_paradoxus	1	Forest	FL1	HW	0.14
Cryptocanthon_paradoxus	1	Forest	FL1	PL	0.13
Cryptocanthon_paradoxus	1	Forest	FL1	PW	0.26
Cryptocanthon_paradoxus	1	Forest	FL1	PH	0.12
Cryptocanthon_paradoxus	1	Forest	FL1	EL	0.23
Cryptocanthon_paradoxus	1	Forest	FL1	pTL	0.13
Cryptocanthon_paradoxus	1	Forest	FL1	pTW	0.03
Cryptocanthon_paradoxus	1	Forest	FL1	mTL	0.13
Cryptocanthon_paradoxus	1	Forest	FL1	Biomass	0.00004
Cryptocanthon_paradoxus	2	Forest	FL1	HL	0.11
Cryptocanthon_paradoxus	2	Forest	FL1	HW	0.15
Cryptocanthon_paradoxus	2	Forest	FL1	PL	0.15
Cryptocanthon_paradoxus	2	Forest	FL1	PW	0.26
Cryptocanthon_paradoxus	2	Forest	FL1	PH	0.12
Cryptocanthon_paradoxus	2	Forest	FL1	EL	0.25
Cryptocanthon_paradoxus	2	Forest	FL1	pTL	0.12
Cryptocanthon_paradoxus	2	Forest	FL1	pTW	0.04
Cryptocanthon_paradoxus	2	Forest	FL1	mTL	0.15
Cryptocanthon_paradoxus	2	Forest	FL1	Biomass	0.00005
Cryptocanthon_paradoxus	3	Forest	FL1	HL	0.09
Cryptocanthon_paradoxus	3	Forest	FL1	HW	0.13
Cryptocanthon_paradoxus	3	Forest	FL1	PL	0.13
Cryptocanthon_paradoxus	3	Forest	FL1	PW	0.20
Cryptocanthon_paradoxus	3	Forest	FL1	PH	0.10
Cryptocanthon_paradoxus	3	Forest	FL1	EL	0.21
Cryptocanthon_paradoxus	3	Forest	FL1	pTL	0.11
Cryptocanthon_paradoxus	3	Forest	FL1	pTW	0.04
Cryptocanthon_paradoxus	3	Forest	FL1	mTL	0.10
Cryptocanthon_paradoxus	3	Forest	FL1	Biomass	0.00003
Cryptocanthon_paradoxus	4	Forest	FL1	HL	0.08
Cryptocanthon_paradoxus	4	Forest	FL1	HW	0.13
Cryptocanthon_paradoxus	4	Forest	FL1	PL	0.13
Cryptocanthon_paradoxus	4	Forest	FL1	PW	0.25
Cryptocanthon_paradoxus	4	Forest	FL1	PH	0.11
Cryptocanthon_paradoxus	4	Forest	FL1	EL	0.26
Cryptocanthon_paradoxus	4	Forest	FL1	pTL	0.09
Cryptocanthon_paradoxus	4	Forest	FL1	pTW	0.03
Cryptocanthon_paradoxus	4	Forest	FL1	mTL	0.15
Cryptocanthon_paradoxus	4	Forest	FL1	Biomass	0.00004
Cryptocanthon_paradoxus	5	Forest	FL1	HL	0.09
Cryptocanthon_paradoxus	5	Forest	FL1	HW	0.13

Cryptocanthon_paradoxus	5	Forest	FL1	PL	0.15
Cryptocanthon_paradoxus	5	Forest	FL1	PW	0.25
Cryptocanthon_paradoxus	5	Forest	FL1	PH	0.10
Cryptocanthon_paradoxus	5	Forest	FL1	EL	0.27
Cryptocanthon_paradoxus	5	Forest	FL1	pTL	0.10
Cryptocanthon_paradoxus	5	Forest	FL1	pTW	0.03
Cryptocanthon_paradoxus	5	Forest	FL1	mTL	0.15
Cryptocanthon_paradoxus	5	Forest	FL1	Biomass	0.00005
Cryptocanthon_paradoxus	6	Forest	FL1	HL	0.08
Cryptocanthon_paradoxus	6	Forest	FL1	HW	0.14
Cryptocanthon_paradoxus	6	Forest	FL1	PL	0.13
Cryptocanthon_paradoxus	6	Forest	FL1	PW	0.20
Cryptocanthon_paradoxus	6	Forest	FL1	PH	0.11
Cryptocanthon_paradoxus	6	Forest	FL1	EL	0.23
Cryptocanthon_paradoxus	6	Forest	FL1	pTL	0.10
Cryptocanthon_paradoxus	6	Forest	FL1	pTW	0.03
Cryptocanthon_paradoxus	6	Forest	FL1	mTL	0.12
Cryptocanthon_paradoxus	6	Forest	FL1	Biomass	0.00003
Cryptocanthon_paradoxus	7	Forest	FL1	HL	0.09
Cryptocanthon_paradoxus	7	Forest	FL1	HW	0.14
Cryptocanthon_paradoxus	7	Forest	FL1	PL	0.14
Cryptocanthon_paradoxus	7	Forest	FL1	PW	0.25
Cryptocanthon_paradoxus	7	Forest	FL1	PH	0.12
Cryptocanthon_paradoxus	7	Forest	FL1	EL	0.28
Cryptocanthon_paradoxus	7	Forest	FL1	pTL	0.12
Cryptocanthon_paradoxus	7	Forest	FL1	pTW	0.03
Cryptocanthon_paradoxus	7	Forest	FL1	mTL	0.12
Cryptocanthon_paradoxus	7	Forest	FL1	Biomass	0.00005
Cryptocanthon_paradoxus	8	Forest	FL1	HL	0.08
Cryptocanthon_paradoxus	8	Forest	FL1	HW	0.14
Cryptocanthon_paradoxus	8	Forest	FL1	PL	0.13
Cryptocanthon_paradoxus	8	Forest	FL1	PW	0.23
Cryptocanthon_paradoxus	8	Forest	FL1	PH	0.14
Cryptocanthon_paradoxus	8	Forest	FL1	EL	0.25
Cryptocanthon_paradoxus	8	Forest	FL1	pTL	0.10
Cryptocanthon_paradoxus	8	Forest	FL1	pTW	0.04
Cryptocanthon_paradoxus	8	Forest	FL1	mTL	0.13
Cryptocanthon_paradoxus	8	Forest	FL1	Biomass	0.00003
Cryptocanthon_paradoxus	9	Forest	FL1	HL	0.09
Cryptocanthon_paradoxus	9	Forest	FL1	HW	0.13
Cryptocanthon_paradoxus	9	Forest	FL1	PL	0.13
Cryptocanthon_paradoxus	9	Forest	FL1	PW	0.25
Cryptocanthon_paradoxus	9	Forest	FL1	PH	0.11

Cryptocanthon_paradoxus	9	Forest	FL1	EL	0.26
Cryptocanthon_paradoxus	9	Forest	FL1	pTL	0.12
Cryptocanthon_paradoxus	9	Forest	FL1	pTW	0.03
Cryptocanthon_paradoxus	9	Forest	FL1	mTL	0.15
Cryptocanthon_paradoxus	9	Forest	FL1	Biomass	0.00004
Cryptocanthon_paradoxus	10	Forest	FL1	HL	0.08
Cryptocanthon_paradoxus	10	Forest	FL1	HW	0.13
Cryptocanthon_paradoxus	10	Forest	FL1	PL	0.14
Cryptocanthon_paradoxus	10	Forest	FL1	PW	0.26
Cryptocanthon_paradoxus	10	Forest	FL1	PH	0.12
Cryptocanthon_paradoxus	10	Forest	FL1	EL	0.28
Cryptocanthon_paradoxus	10	Forest	FL1	pTL	0.13
Cryptocanthon_paradoxus	10	Forest	FL1	pTW	0.03
Cryptocanthon_paradoxus	10	Forest	FL1	mTL	0.12
Cryptocanthon_paradoxus	10	Forest	FL1	Biomass	0.00004
Onoreidium_cristatum	1	Pinus	PL2	HL	0.07
Onoreidium_cristatum	1	Pinus	PL2	HW	0.12
Onoreidium_cristatum	1	Pinus	PL2	PL	0.13
Onoreidium_cristatum	1	Pinus	PL2	PW	0.22
Onoreidium_cristatum	1	Pinus	PL2	PH	0.10
Onoreidium_cristatum	1	Pinus	PL2	EL	0.28
Onoreidium_cristatum	1	Pinus	PL2	pTL	0.07
Onoreidium_cristatum	1	Pinus	PL2	pTW	0.03
Onoreidium_cristatum	1	Pinus	PL2	mTL	0.10
Onoreidium_cristatum	1	Pinus	PL2	Biomass	0.00004
Onoreidium_cristatum	2	Eucalyptus	EL2	HL	0.08
Onoreidium_cristatum	2	Eucalyptus	EL2	HW	0.12
Onoreidium_cristatum	2	Eucalyptus	EL2	PL	0.14
Onoreidium_cristatum	2	Eucalyptus	EL2	PW	0.22
Onoreidium_cristatum	2	Eucalyptus	EL2	PH	0.10
Onoreidium_cristatum	2	Eucalyptus	EL2	EL	0.29
Onoreidium_cristatum	2	Eucalyptus	EL2	pTL	0.08
Onoreidium_cristatum	2	Eucalyptus	EL2	pTW	0.03
Onoreidium_cristatum	2	Eucalyptus	EL2	mTL	0.11
Onoreidium_cristatum	2	Eucalyptus	EL2	Biomass	0.00005
Deltochilum_robustus	1	Pinus	PL2	HL	0.42
Deltochilum_robustus	1	Pinus	PL2	HW	0.75
Deltochilum_robustus	1	Pinus	PL2	PL	0.70
Deltochilum_robustus	1	Pinus	PL2	PW	1.20
Deltochilum_robustus	1	Pinus	PL2	PH	0.60
Deltochilum_robustus	1	Pinus	PL2	EL	1.25
Deltochilum_robustus	1	Pinus	PL2	pTL	0.55
Deltochilum_robustus	1	Pinus	PL2	pTW	0.19

Deltochilum_robustus	1	Pinus	PL2	mTL	0.70
Deltochilum_robustus	1	Pinus	PL2	Biomass	0.00292
Deltochilum_robustus	2	Pinus	PL2	HL	0.35
Deltochilum_robustus	2	Pinus	PL2	HW	0.70
Deltochilum_robustus	2	Pinus	PL2	PL	0.68
Deltochilum_robustus	2	Pinus	PL2	PW	1.15
Deltochilum_robustus	2	Pinus	PL2	PH	0.65
Deltochilum_robustus	2	Pinus	PL2	EL	1.18
Deltochilum_robustus	2	Pinus	PL2	pTL	0.47
Deltochilum_robustus	2	Pinus	PL2	pTW	0.17
Deltochilum_robustus	2	Pinus	PL2	mTL	0.65
Deltochilum_robustus	2	Pinus	PL2	Biomass	0.00243
Ateuchus_aenomicans	1	Forest	FL2	HL	0.16
Ateuchus_aenomicans	1	Forest	FL2	HW	0.19
Ateuchus_aenomicans	1	Forest	FL2	PL	0.26
Ateuchus_aenomicans	1	Forest	FL2	PW	0.33
Ateuchus_aenomicans	1	Forest	FL2	PH	0.15
Ateuchus_aenomicans	1	Forest	FL2	EL	0.36
Ateuchus_aenomicans	1	Forest	FL2	pTL	0.10
Ateuchus_aenomicans	1	Forest	FL2	pTW	0.06
Ateuchus_aenomicans	1	Forest	FL2	mTL	0.16
Ateuchus_aenomicans	1	Forest	FL2	Biomass	0.00015
Ateuchus_aenomicans	2	Forest	FL2	HL	0.12
Ateuchus_aenomicans	2	Forest	FL2	HW	0.20
Ateuchus_aenomicans	2	Forest	FL2	PL	0.22
Ateuchus_aenomicans	2	Forest	FL2	PW	0.35
Ateuchus_aenomicans	2	Forest	FL2	PH	0.15
Ateuchus_aenomicans	2	Forest	FL2	EL	0.38
Ateuchus_aenomicans	2	Forest	FL2	pTL	0.11
Ateuchus_aenomicans	2	Forest	FL2	pTW	0.05
Ateuchus_aenomicans	2	Forest	FL2	mTL	0.16
Ateuchus_aenomicans	2	Forest	FL2	Biomass	0.00012
Ateuchus_aenomicans	3	Forest	FL2	HL	0.12
Ateuchus_aenomicans	3	Forest	FL2	HW	0.19
Ateuchus_aenomicans	3	Forest	FL2	PL	0.25
Ateuchus_aenomicans	3	Forest	FL2	PW	0.35
Ateuchus_aenomicans	3	Forest	FL2	PH	0.16
Ateuchus_aenomicans	3	Forest	FL2	EL	0.38
Ateuchus_aenomicans	3	Forest	FL2	pTL	0.11
Ateuchus_aenomicans	3	Forest	FL2	pTW	0.06
Ateuchus_aenomicans	3	Forest	FL2	mTL	0.15
Ateuchus_aenomicans	3	Forest	FL2	Biomass	0.00014
Ateuchus_aenomicans	4	Forest	FL2	HL	0.15

Ateuchus_aenomicans	4	Forest	FL2	HW	0.21
Ateuchus_aenomicans	4	Forest	FL2	PL	0.23
Ateuchus_aenomicans	4	Forest	FL2	PW	0.36
Ateuchus_aenomicans	4	Forest	FL2	PH	0.16
Ateuchus_aenomicans	4	Forest	FL2	EL	0.35
Ateuchus_aenomicans	4	Forest	FL2	pTL	0.10
Ateuchus_aenomicans	4	Forest	FL2	pTW	0.06
Ateuchus_aenomicans	4	Forest	FL2	mTL	0.15
Ateuchus_aenomicans	4	Forest	FL2	Biomass	0.00013
Ateuchus_aenomicans	5	Forest	FL2	HL	0.14
Ateuchus_aenomicans	5	Forest	FL2	HW	0.21
Ateuchus_aenomicans	5	Forest	FL2	PL	0.25
Ateuchus_aenomicans	5	Forest	FL2	PW	0.34
Ateuchus_aenomicans	5	Forest	FL2	PH	0.15
Ateuchus_aenomicans	5	Forest	FL2	EL	0.36
Ateuchus_aenomicans	5	Forest	FL2	pTL	0.13
Ateuchus_aenomicans	5	Forest	FL2	pTW	0.05
Ateuchus_aenomicans	5	Forest	FL2	mTL	0.15
Ateuchus_aenomicans	5	Forest	FL2	Biomass	0.00014
Ateuchus_aenomicans	6	Forest	FL2	HL	0.13
Ateuchus_aenomicans	6	Forest	FL2	HW	0.20
Ateuchus_aenomicans	6	Forest	FL2	PL	0.25
Ateuchus_aenomicans	6	Forest	FL2	PW	0.33
Ateuchus_aenomicans	6	Forest	FL2	PH	0.14
Ateuchus_aenomicans	6	Forest	FL2	EL	0.34
Ateuchus_aenomicans	6	Forest	FL2	pTL	0.15
Ateuchus_aenomicans	6	Forest	FL2	pTW	0.06
Ateuchus_aenomicans	6	Forest	FL2	mTL	0.14
Ateuchus_aenomicans	6	Forest	FL2	Biomass	0.00012
Ateuchus_aenomicans	7	Forest	FL2	HL	0.12
Ateuchus_aenomicans	7	Forest	FL2	HW	0.21
Ateuchus_aenomicans	7	Forest	FL2	PL	0.23
Ateuchus_aenomicans	7	Forest	FL2	PW	0.34
Ateuchus_aenomicans	7	Forest	FL2	PH	0.13
Ateuchus_aenomicans	7	Forest	FL2	EL	0.38
Ateuchus_aenomicans	7	Forest	FL2	pTL	0.12
Ateuchus_aenomicans	7	Forest	FL2	pTW	0.05
Ateuchus_aenomicans	7	Forest	FL2	mTL	0.13
Ateuchus_aenomicans	7	Forest	FL2	Biomass	0.00013
Ateuchus_aenomicans	8	Forest	FL2	HL	0.15
Ateuchus_aenomicans	8	Forest	FL2	HW	0.20
Ateuchus_aenomicans	8	Forest	FL2	PL	0.26
Ateuchus_aenomicans	8	Forest	FL2	PW	0.38

Ateuchus_aenomicans	8	Forest	FL2	PH	0.18
Ateuchus_aenomicans	8	Forest	FL2	EL	0.39
Ateuchus_aenomicans	8	Forest	FL2	pTL	0.15
Ateuchus_aenomicans	8	Forest	FL2	pTW	0.07
Ateuchus_aenomicans	8	Forest	FL2	mTL	0.15
Ateuchus_aenomicans	8	Forest	FL2	Biomass	0.00016
Ateuchus_aenomicans	9	Forest	FL2	HL	0.18
Ateuchus_aenomicans	9	Forest	FL2	HW	0.21
Ateuchus_aenomicans	9	Forest	FL2	PL	0.25
Ateuchus_aenomicans	9	Forest	FL2	PW	0.33
Ateuchus_aenomicans	9	Forest	FL2	PH	0.20
Ateuchus_aenomicans	9	Forest	FL2	EL	0.38
Ateuchus_aenomicans	9	Forest	FL2	pTL	0.15
Ateuchus_aenomicans	9	Forest	FL2	pTW	0.05
Ateuchus_aenomicans	9	Forest	FL2	mTL	0.14
Ateuchus_aenomicans	9	Forest	FL2	Biomass	0.00017
Ateuchus_aenomicans	10	Forest	FL2	HL	0.16
Ateuchus_aenomicans	10	Forest	FL2	HW	0.20
Ateuchus_aenomicans	10	Forest	FL2	PL	0.23
Ateuchus_aenomicans	10	Forest	FL2	PW	0.35
Ateuchus_aenomicans	10	Forest	FL2	PH	0.17
Ateuchus_aenomicans	10	Forest	FL2	EL	0.35
Ateuchus_aenomicans	10	Forest	FL2	pTL	0.12
Ateuchus_aenomicans	10	Forest	FL2	pTW	0.06
Ateuchus_aenomicans	10	Forest	FL2	mTL	0.15
Ateuchus_aenomicans	10	Forest	FL2	Biomass	0.00013
Uroxys_sp1	1	Forest	FL2	HL	0.18
Uroxys_sp1	1	Forest	FL2	HW	0.30
Uroxys_sp1	1	Forest	FL2	PL	0.27
Uroxys_sp1	1	Forest	FL2	PW	0.41
Uroxys_sp1	1	Forest	FL2	PH	0.20
Uroxys_sp1	1	Forest	FL2	EL	0.57
Uroxys_sp1	1	Forest	FL2	pTL	0.22
Uroxys_sp1	1	Forest	FL2	pTW	0.07
Uroxys_sp1	1	Forest	FL2	mTL	0.20
Uroxys_sp1	1	Forest	FL2	Biomass	0.00032
Uroxys_sp1	2	Forest	FL2	HL	0.19
Uroxys_sp1	2	Forest	FL2	HW	0.30
Uroxys_sp1	2	Forest	FL2	PL	0.31
Uroxys_sp1	2	Forest	FL2	PW	0.51
Uroxys_sp1	2	Forest	FL2	PH	0.21
Uroxys_sp1	2	Forest	FL2	EL	0.56
Uroxys_sp1	2	Forest	FL2	pTL	0.26

Uroxys_sp1	2	Forest	FL2	pTW	0.06
Uroxys_sp1	2	Forest	FL2	mTL	0.24
Uroxys_sp1	2	Forest	FL2	Biomass	0.00035
Uroxys_sp1	3	Forest	FL2	HL	0.15
Uroxys_sp1	3	Forest	FL2	HW	0.24
Uroxys_sp1	3	Forest	FL2	PL	0.23
Uroxys_sp1	3	Forest	FL2	PW	0.40
Uroxys_sp1	3	Forest	FL2	PH	0.19
Uroxys_sp1	3	Forest	FL2	EL	0.49
Uroxys_sp1	3	Forest	FL2	pTL	0.18
Uroxys_sp1	3	Forest	FL2	pTW	0.04
Uroxys_sp1	3	Forest	FL2	mTL	0.20
Uroxys_sp1	3	Forest	FL2	Biomass	0.00021
Uroxys_sp1	4	Forest	FL2	HL	0.18
Uroxys_sp1	4	Forest	FL2	HW	0.3
Uroxys_sp1	4	Forest	FL2	PL	0.32
Uroxys_sp1	4	Forest	FL2	PW	0.51
Uroxys_sp1	4	Forest	FL2	PH	0.21
Uroxys_sp1	4	Forest	FL2	EL	0.65
Uroxys_sp1	4	Forest	FL2	pTL	0.28
Uroxys_sp1	4	Forest	FL2	pTW	0.07
Uroxys_sp1	4	Forest	FL2	mTL	0.23
Uroxys_sp1	4	Forest	FL2	Biomass	0.00043
Uroxys_sp1	5	Forest	FL2	HL	0.19
Uroxys_sp1	5	Forest	FL2	HW	0.30
Uroxys_sp1	5	Forest	FL2	PL	0.35
Uroxys_sp1	5	Forest	FL2	PW	0.55
Uroxys_sp1	5	Forest	FL2	PH	0.23
Uroxys_sp1	5	Forest	FL2	EL	0.63
Uroxys_sp1	5	Forest	FL2	pTL	0.32
Uroxys_sp1	5	Forest	FL2	pTW	0.08
Uroxys_sp1	5	Forest	FL2	mTL	0.25
Uroxys_sp1	5	Forest	FL2	Biomass	0.00046
Uroxys_sp1	6	Forest	FL2	HL	0.19
Uroxys_sp1	6	Forest	FL2	HW	0.30
Uroxys_sp1	6	Forest	FL2	PL	0.36
Uroxys_sp1	6	Forest	FL2	PW	0.54
Uroxys_sp1	6	Forest	FL2	PH	0.22
Uroxys_sp1	6	Forest	FL2	EL	0.65
Uroxys_sp1	6	Forest	FL2	pTL	0.35
Uroxys_sp1	6	Forest	FL2	pTW	0.08
Uroxys_sp1	6	Forest	FL2	mTL	0.24
Uroxys_sp1	6	Forest	FL2	Biomass	0.00049

Uroxys_sp1	7	Forest	FL2	HL	0.15
Uroxys_sp1	7	Forest	FL2	HW	0.26
Uroxys_sp1	7	Forest	FL2	PL	0.30
Uroxys_sp1	7	Forest	FL2	PW	0.49
Uroxys_sp1	7	Forest	FL2	PH	0.21
Uroxys_sp1	7	Forest	FL2	EL	0.59
Uroxys_sp1	7	Forest	FL2	pTL	0.30
Uroxys_sp1	7	Forest	FL2	pTW	0.06
Uroxys_sp1	7	Forest	FL2	mTL	0.22
Uroxys_sp1	7	Forest	FL2	Biomass	0.00033
Uroxys_sp1	8	Forest	FL2	HL	0.12
Uroxys_sp1	8	Forest	FL2	HW	0.28
Uroxys_sp1	8	Forest	FL2	PL	0.31
Uroxys_sp1	8	Forest	FL2	PW	0.48
Uroxys_sp1	8	Forest	FL2	PH	0.19
Uroxys_sp1	8	Forest	FL2	EL	0.56
Uroxys_sp1	8	Forest	FL2	pTL	0.29
Uroxys_sp1	8	Forest	FL2	pTW	0.06
Uroxys_sp1	8	Forest	FL2	mTL	0.21
Uroxys_sp1	8	Forest	FL2	Biomass	0.00029
Uroxys_sp1	9	Forest	FL2	HL	0.10
Uroxys_sp1	9	Forest	FL2	HW	0.20
Uroxys_sp1	9	Forest	FL2	PL	0.20
Uroxys_sp1	9	Forest	FL2	PW	0.34
Uroxys_sp1	9	Forest	FL2	PH	0.18
Uroxys_sp1	9	Forest	FL2	EL	0.49
Uroxys_sp1	9	Forest	FL2	pTL	0.15
Uroxys_sp1	9	Forest	FL2	pTW	0.04
Uroxys_sp1	9	Forest	FL2	mTL	0.19
Uroxys_sp1	9	Forest	FL2	Biomass	0.00016
Uroxys_sp1	10	Forest	FL2	HL	0.19
Uroxys_sp1	10	Forest	FL2	HW	0.30
Uroxys_sp1	10	Forest	FL2	PL	0.30
Uroxys_sp1	10	Forest	FL2	PW	0.50
Uroxys_sp1	10	Forest	FL2	PH	0.23
Uroxys_sp1	10	Forest	FL2	EL	0.56
Uroxys_sp1	10	Forest	FL2	pTL	0.22
Uroxys_sp1	10	Forest	FL2	pTW	0.12
Uroxys_sp1	10	Forest	FL2	mTL	0.22
Uroxys_sp1	10	Forest	FL2	Biomass	0.00034
Uroxys_sp1	1	Forest	FL1	HL	0.14
Uroxys_sp1	1	Forest	FL1	HW	0.20
Uroxys_sp1	1	Forest	FL1	PL	0.30

Uroxys_sp1	1	Forest	FL1	PW	0.51
Uroxys_sp1	1	Forest	FL1	PH	0.20
Uroxys_sp1	1	Forest	FL1	EL	0.65
Uroxys_sp1	1	Forest	FL1	pTL	0.22
Uroxys_sp1	1	Forest	FL1	pTW	0.06
Uroxys_sp1	1	Forest	FL1	mTL	0.23
Uroxys_sp1	1	Forest	FL1	Biomass	0.00038
Uroxys_sp1	2	Forest	FL1	HL	0.14
Uroxys_sp1	2	Forest	FL1	HW	0.24
Uroxys_sp1	2	Forest	FL1	PL	0.25
Uroxys_sp1	2	Forest	FL1	PW	0.45
Uroxys_sp1	2	Forest	FL1	PH	0.19
Uroxys_sp1	2	Forest	FL1	EL	0.58
Uroxys_sp1	2	Forest	FL1	pTL	0.20
Uroxys_sp1	2	Forest	FL1	pTW	0.05
Uroxys_sp1	2	Forest	FL1	mTL	0.22
Uroxys_sp1	2	Forest	FL1	Biomass	0.00028
Uroxys_sp1	3	Forest	FL1	HL	0.18
Uroxys_sp1	3	Forest	FL1	HW	0.30
Uroxys_sp1	3	Forest	FL1	PL	0.28
Uroxys_sp1	3	Forest	FL1	PW	0.50
Uroxys_sp1	3	Forest	FL1	PH	0.21
Uroxys_sp1	3	Forest	FL1	EL	0.56
Uroxys_sp1	3	Forest	FL1	pTL	0.22
Uroxys_sp1	3	Forest	FL1	pTW	0.07
Uroxys_sp1	3	Forest	FL1	mTL	0.24
Uroxys_sp1	3	Forest	FL1	Biomass	0.00032
Uroxys_sp1	4	Forest	FL1	HL	0.24
Uroxys_sp1	4	Forest	FL1	HW	0.29
Uroxys_sp1	4	Forest	FL1	PL	0.36
Uroxys_sp1	4	Forest	FL1	PW	0.60
Uroxys_sp1	4	Forest	FL1	PH	0.23
Uroxys_sp1	4	Forest	FL1	EL	0.67
Uroxys_sp1	4	Forest	FL1	pTL	0.40
Uroxys_sp1	4	Forest	FL1	pTW	0.08
Uroxys_sp1	4	Forest	FL1	mTL	0.26
Uroxys_sp1	4	Forest	FL1	Biomass	0.00057
Uroxys_sp1	5	Forest	FL1	HL	0.20
Uroxys_sp1	5	Forest	FL1	HW	0.30
Uroxys_sp1	5	Forest	FL1	PL	0.29
Uroxys_sp1	5	Forest	FL1	PW	0.52
Uroxys_sp1	5	Forest	FL1	PH	0.24
Uroxys_sp1	5	Forest	FL1	EL	0.54

Uroxys_sp1	5	Forest	FL1	pTL	0.24
Uroxys_sp1	5	Forest	FL1	pTW	0.12
Uroxys_sp1	5	Forest	FL1	mTL	0.22
Uroxys_sp1	5	Forest	FL1	Biomass	0.00032
Uroxys_sp1	1	Pinus	PL1	HL	0.17
Uroxys_sp1	1	Pinus	PL1	HW	0.27
Uroxys_sp1	1	Pinus	PL1	PL	0.28
Uroxys_sp1	1	Pinus	PL1	PW	0.45
Uroxys_sp1	1	Pinus	PL1	PH	0.22
Uroxys_sp1	1	Pinus	PL1	EL	0.48
Uroxys_sp1	1	Pinus	PL1	pTL	0.22
Uroxys_sp1	1	Pinus	PL1	pTW	0.06
Uroxys_sp1	1	Pinus	PL1	mTL	0.20
Uroxys_sp1	1	Pinus	PL1	Biomass	0.00025
Uroxys_sp1	2	Pinus	PL1	HL	0.15
Uroxys_sp1	2	Pinus	PL1	HW	0.26
Uroxys_sp1	2	Pinus	PL1	PL	0.22
Uroxys_sp1	2	Pinus	PL1	PW	0.39
Uroxys_sp1	2	Pinus	PL1	PH	0.21
Uroxys_sp1	2	Pinus	PL1	EL	0.40
Uroxys_sp1	2	Pinus	PL1	pTL	0.18
Uroxys_sp1	2	Pinus	PL1	pTW	0.05
Uroxys_sp1	2	Pinus	PL1	mTL	0.22
Uroxys_sp1	2	Pinus	PL1	Biomass	0.00015
Uroxys_sp1	3	Pinus	PL1	HL	0.20
Uroxys_sp1	3	Pinus	PL1	HW	0.28
Uroxys_sp1	3	Pinus	PL1	PL	0.27
Uroxys_sp1	3	Pinus	PL1	PW	0.46
Uroxys_sp1	3	Pinus	PL1	PH	0.21
Uroxys_sp1	3	Pinus	PL1	EL	0.52
Uroxys_sp1	3	Pinus	PL1	pTL	0.21
Uroxys_sp1	3	Pinus	PL1	pTW	0.07
Uroxys_sp1	3	Pinus	PL1	mTL	0.21
Uroxys_sp1	3	Pinus	PL1	Biomass	0.00029
Uroxys_sp1	4	Pinus	PL1	HL	0.16
Uroxys_sp1	4	Pinus	PL1	HW	0.25
Uroxys_sp1	4	Pinus	PL1	PL	0.21
Uroxys_sp1	4	Pinus	PL1	PW	0.40
Uroxys_sp1	4	Pinus	PL1	PH	0.19
Uroxys_sp1	4	Pinus	PL1	EL	0.43
Uroxys_sp1	4	Pinus	PL1	pTL	0.20
Uroxys_sp1	4	Pinus	PL1	pTW	0.06
Uroxys_sp1	4	Pinus	PL1	mTL	0.16

Uroxys_sp1	4	Pinus	PL1	Biomass	0.00016
Uroxys_sp1	5	Pinus	PL1	HL	0.16
Uroxys_sp1	5	Pinus	PL1	HW	0.30
Uroxys_sp1	5	Pinus	PL1	PL	0.30
Uroxys_sp1	5	Pinus	PL1	PW	0.50
Uroxys_sp1	5	Pinus	PL1	PH	0.21
Uroxys_sp1	5	Pinus	PL1	EL	0.52
Uroxys_sp1	5	Pinus	PL1	pTL	0.29
Uroxys_sp1	5	Pinus	PL1	pTW	0.07
Uroxys_sp1	5	Pinus	PL1	mTL	0.29
Uroxys_sp1	5	Pinus	PL1	Biomass	0.00028
Uroxys_sp1	6	Pinus	PL1	HL	0.20
Uroxys_sp1	6	Pinus	PL1	HW	0.30
Uroxys_sp1	6	Pinus	PL1	PL	0.23
Uroxys_sp1	6	Pinus	PL1	PW	0.55
Uroxys_sp1	6	Pinus	PL1	PH	0.20
Uroxys_sp1	6	Pinus	PL1	EL	0.55
Uroxys_sp1	6	Pinus	PL1	pTL	0.24
Uroxys_sp1	6	Pinus	PL1	pTW	0.10
Uroxys_sp1	6	Pinus	PL1	mTL	0.25
Uroxys_sp1	6	Pinus	PL1	Biomass	0.00028
Uroxys_sp1	7	Pinus	PL1	HL	0.19
Uroxys_sp1	7	Pinus	PL1	HW	0.28
Uroxys_sp1	7	Pinus	PL1	PL	0.29
Uroxys_sp1	7	Pinus	PL1	PW	0.49
Uroxys_sp1	7	Pinus	PL1	PH	0.20
Uroxys_sp1	7	Pinus	PL1	EL	0.52
Uroxys_sp1	7	Pinus	PL1	pTL	0.3
Uroxys_sp1	7	Pinus	PL1	pTW	0.07
Uroxys_sp1	7	Pinus	PL1	mTL	0.18
Uroxys_sp1	7	Pinus	PL1	Biomass	0.000305
Uroxys_sp1	8	Pinus	PL1	HL	0.16
Uroxys_sp1	8	Pinus	PL1	HW	0.29
Uroxys_sp1	8	Pinus	PL1	PL	0.26
Uroxys_sp1	8	Pinus	PL1	PW	0.46
Uroxys_sp1	8	Pinus	PL1	PH	0.2
Uroxys_sp1	8	Pinus	PL1	EL	0.47
Uroxys_sp1	8	Pinus	PL1	pTL	0.19
Uroxys_sp1	8	Pinus	PL1	pTW	0.06
Uroxys_sp1	8	Pinus	PL1	mTL	0.22
Uroxys_sp1	8	Pinus	PL1	Biomass	0.00022
Uroxys_sp1	9	Pinus	PL1	HL	0.2
Uroxys_sp1	9	Pinus	PL1	HW	0.29

Uroxys_sp1	9	Pinus	PL1	PL	0.26
Uroxys_sp1	9	Pinus	PL1	PW	0.45
Uroxys_sp1	9	Pinus	PL1	PH	0.3
Uroxys_sp1	9	Pinus	PL1	EL	0.47
Uroxys_sp1	9	Pinus	PL1	pTL	0.18
Uroxys_sp1	9	Pinus	PL1	pTW	0.07
Uroxys_sp1	9	Pinus	PL1	mTL	0.2
Uroxys_sp1	9	Pinus	PL1	Biomass	0.00025
Uroxys_sp1	10	Pinus	PL1	HL	0.19
Uroxys_sp1	10	Pinus	PL1	HW	0.28
Uroxys_sp1	10	Pinus	PL1	PL	0.26
Uroxys_sp1	10	Pinus	PL1	PW	0.45
Uroxys_sp1	10	Pinus	PL1	PH	0.27
Uroxys_sp1	10	Pinus	PL1	EL	0.47
Uroxys_sp1	10	Pinus	PL1	pTL	0.15
Uroxys_sp1	10	Pinus	PL1	pTW	0.05
Uroxys_sp1	10	Pinus	PL1	mTL	0.17
Uroxys_sp1	10	Pinus	PL1	Biomass	0.00024
Uroxys_sp1	1	Pinus	PL2	HL	0.2
Uroxys_sp1	1	Pinus	PL2	HW	0.3
Uroxys_sp1	1	Pinus	PL2	PL	0.33
Uroxys_sp1	1	Pinus	PL2	PW	0.52
Uroxys_sp1	1	Pinus	PL2	PH	0.3
Uroxys_sp1	1	Pinus	PL2	EL	0.55
Uroxys_sp1	1	Pinus	PL2	pTL	0.3
Uroxys_sp1	1	Pinus	PL2	pTW	0.07
Uroxys_sp1	1	Pinus	PL2	mTL	0.22
Uroxys_sp1	1	Pinus	PL2	Biomass	0.00037
Uroxys_sp1	2	Pinus	PL2	HL	0.2
Uroxys_sp1	2	Pinus	PL2	HW	0.16
Uroxys_sp1	2	Pinus	PL2	PL	0.26
Uroxys_sp1	2	Pinus	PL2	PW	0.46
Uroxys_sp1	2	Pinus	PL2	PH	0.15
Uroxys_sp1	2	Pinus	PL2	EL	0.45
Uroxys_sp1	2	Pinus	PL2	pTL	0.3
Uroxys_sp1	2	Pinus	PL2	pTW	0.07
Uroxys_sp1	2	Pinus	PL2	mTL	0.15
Uroxys_sp1	2	Pinus	PL2	Biomass	0.00023
Uroxys_sp1	3	Pinus	PL2	HL	0.16
Uroxys_sp1	3	Pinus	PL2	HW	0.3
Uroxys_sp1	3	Pinus	PL2	PL	0.34
Uroxys_sp1	3	Pinus	PL2	PW	0.5
Uroxys_sp1	3	Pinus	PL2	PH	0.22

Uroxys_sp1	3	Pinus	PL2	EL	0.58
Uroxys_sp1	3	Pinus	PL2	pTL	0.3
Uroxys_sp1	3	Pinus	PL2	pTW	0.12
Uroxys_sp1	3	Pinus	PL2	mTL	0.22
Uroxys_sp1	3	Pinus	PL2	Biomass	0.00037
Uroxys_sp1	4	Pinus	PL2	HL	0.12
Uroxys_sp1	4	Pinus	PL2	HW	0.28
Uroxys_sp1	4	Pinus	PL2	PL	0.33
Uroxys_sp1	4	Pinus	PL2	PW	0.48
Uroxys_sp1	4	Pinus	PL2	PH	0.2
Uroxys_sp1	4	Pinus	PL2	EL	0.58
Uroxys_sp1	4	Pinus	PL2	pTL	0.25
Uroxys_sp1	4	Pinus	PL2	pTW	0.1
Uroxys_sp1	4	Pinus	PL2	mTL	0.19
Uroxys_sp1	4	Pinus	PL2	Biomass	0.00032
Uroxys_sp1	5	Pinus	PL2	HL	0.2
Uroxys_sp1	5	Pinus	PL2	HW	0.32
Uroxys_sp1	5	Pinus	PL2	PL	0.33
Uroxys_sp1	5	Pinus	PL2	PW	0.56
Uroxys_sp1	5	Pinus	PL2	PH	0.22
Uroxys_sp1	5	Pinus	PL2	EL	0.67
Uroxys_sp1	5	Pinus	PL2	pTL	0.23
Uroxys_sp1	5	Pinus	PL2	pTW	0.09
Uroxys_sp1	5	Pinus	PL2	mTL	0.29
Uroxys_sp1	5	Pinus	PL2	Biomass	0.00049
Uroxys_sp1	6	Pinus	PL2	HL	0.15
Uroxys_sp1	6	Pinus	PL2	HW	0.26
Uroxys_sp1	6	Pinus	PL2	PL	0.22
Uroxys_sp1	6	Pinus	PL2	PW	0.38
Uroxys_sp1	6	Pinus	PL2	PH	0.25
Uroxys_sp1	6	Pinus	PL2	EL	0.4
Uroxys_sp1	6	Pinus	PL2	pTL	0.19
Uroxys_sp1	6	Pinus	PL2	pTW	0.06
Uroxys_sp1	6	Pinus	PL2	mTL	0.16
Uroxys_sp1	6	Pinus	PL2	Biomass	0.00015
Uroxys_sp1	7	Pinus	PL2	HL	0.18
Uroxys_sp1	7	Pinus	PL2	HW	0.29
Uroxys_sp1	7	Pinus	PL2	PL	0.26
Uroxys_sp1	7	Pinus	PL2	PW	0.44
Uroxys_sp1	7	Pinus	PL2	PH	0.20
Uroxys_sp1	7	Pinus	PL2	EL	0.50
Uroxys_sp1	7	Pinus	PL2	pTL	0.22
Uroxys_sp1	7	Pinus	PL2	pTW	0.08

Uroxys_sp1	7	Pinus	PL2	mTL	0.22
Uroxys_sp1	7	Pinus	PL2	Biomass	0.00025
Uroxys_sp1	8	Pinus	PL2	HL	0.12
Uroxys_sp1	8	Pinus	PL2	HW	0.31
Uroxys_sp1	8	Pinus	PL2	PL	0.29
Uroxys_sp1	8	Pinus	PL2	PW	0.47
Uroxys_sp1	8	Pinus	PL2	PH	0.18
Uroxys_sp1	8	Pinus	PL2	EL	0.53
Uroxys_sp1	8	Pinus	PL2	pTL	0.19
Uroxys_sp1	8	Pinus	PL2	pTW	0.10
Uroxys_sp1	8	Pinus	PL2	mTL	0.25
Uroxys_sp1	8	Pinus	PL2	Biomass	0.00025
Uroxys_sp1	9	Pinus	PL2	HL	0.12
Uroxys_sp1	9	Pinus	PL2	HW	0.27
Uroxys_sp1	9	Pinus	PL2	PL	0.23
Uroxys_sp1	9	Pinus	PL2	PW	0.4
Uroxys_sp1	9	Pinus	PL2	PH	0.17
Uroxys_sp1	9	Pinus	PL2	EL	0.4
Uroxys_sp1	9	Pinus	PL2	pTL	0.17
Uroxys_sp1	9	Pinus	PL2	pTW	0.07
Uroxys_sp1	9	Pinus	PL2	mTL	0.22
Uroxys_sp1	9	Pinus	PL2	Biomass	0.00014
Uroxys_sp1	10	Pinus	PL2	HL	0.16
Uroxys_sp1	10	Pinus	PL2	HW	0.28
Uroxys_sp1	10	Pinus	PL2	PL	0.25
Uroxys_sp1	10	Pinus	PL2	PW	0.44
Uroxys_sp1	10	Pinus	PL2	PH	0.24
Uroxys_sp1	10	Pinus	PL2	EL	0.47
Uroxys_sp1	10	Pinus	PL2	pTL	0.17
Uroxys_sp1	10	Pinus	PL2	pTW	0.08
Uroxys_sp1	10	Pinus	PL2	mTL	0.17
Uroxys_sp1	10	Pinus	PL2	Biomass	0.00021
Uroxys_sp1	1	Eucalyptus	EL2	HL	0.18
Uroxys_sp1	1	Eucalyptus	EL2	HW	0.26
Uroxys_sp1	1	Eucalyptus	EL2	PL	0.22
Uroxys_sp1	1	Eucalyptus	EL2	PW	0.42
Uroxys_sp1	1	Eucalyptus	EL2	PH	0.3
Uroxys_sp1	1	Eucalyptus	EL2	EL	0.43
Uroxys_sp1	1	Eucalyptus	EL2	pTL	0.18
Uroxys_sp1	1	Eucalyptus	EL2	pTW	0.04
Uroxys_sp1	1	Eucalyptus	EL2	mTL	0.2
Uroxys_sp1	1	Eucalyptus	EL2	Biomass	0.00018
Uroxys_sp1	2	Eucalyptus	EL2	HL	0.22

Uroxys_sp1	2	Eucalyptus	EL2	HW	0.31
Uroxys_sp1	2	Eucalyptus	EL2	PL	0.26
Uroxys_sp1	2	Eucalyptus	EL2	PW	0.49
Uroxys_sp1	2	Eucalyptus	EL2	PH	0.35
Uroxys_sp1	2	Eucalyptus	EL2	EL	0.52
Uroxys_sp1	2	Eucalyptus	EL2	pTL	0.18
Uroxys_sp1	2	Eucalyptus	EL2	pTW	0.07
Uroxys_sp1	2	Eucalyptus	EL2	mTL	0.22
Uroxys_sp1	2	Eucalyptus	EL2	Biomass	0.00018
Uroxys_sp1	3	Eucalyptus	EL2	HL	0.15
Uroxys_sp1	3	Eucalyptus	EL2	HW	0.29
Uroxys_sp1	3	Eucalyptus	EL2	PL	0.25
Uroxys_sp1	3	Eucalyptus	EL2	PW	0.45
Uroxys_sp1	3	Eucalyptus	EL2	PH	0.29
Uroxys_sp1	3	Eucalyptus	EL2	EL	0.42
Uroxys_sp1	3	Eucalyptus	EL2	pTL	0.16
Uroxys_sp1	3	Eucalyptus	EL2	pTW	0.06
Uroxys_sp1	3	Eucalyptus	EL2	mTL	0.19
Uroxys_sp1	3	Eucalyptus	EL2	Biomass	0.00018
Uroxys_sp1	4	Eucalyptus	EL2	HL	0.2
Uroxys_sp1	4	Eucalyptus	EL2	HW	0.3
Uroxys_sp1	4	Eucalyptus	EL2	PL	0.25
Uroxys_sp1	4	Eucalyptus	EL2	PW	0.43
Uroxys_sp1	4	Eucalyptus	EL2	PH	0.3
Uroxys_sp1	4	Eucalyptus	EL2	EL	0.5
Uroxys_sp1	4	Eucalyptus	EL2	pTL	0.18
Uroxys_sp1	4	Eucalyptus	EL2	pTW	0.06
Uroxys_sp1	4	Eucalyptus	EL2	mTL	0.2
Uroxys_sp1	4	Eucalyptus	EL2	Biomass	0.00026
Uroxys_sp1	5	Eucalyptus	EL2	HL	0.23
Uroxys_sp1	5	Eucalyptus	EL2	HW	0.3
Uroxys_sp1	5	Eucalyptus	EL2	PL	0.24
Uroxys_sp1	5	Eucalyptus	EL2	PW	0.45
Uroxys_sp1	5	Eucalyptus	EL2	PH	0.31
Uroxys_sp1	5	Eucalyptus	EL2	EL	0.52
Uroxys_sp1	5	Eucalyptus	EL2	pTL	0.18
Uroxys_sp1	5	Eucalyptus	EL2	pTW	0.06
Uroxys_sp1	5	Eucalyptus	EL2	mTL	0.2
Uroxys_sp1	5	Eucalyptus	EL2	Biomass	0.00029
Uroxys_sp1	6	Eucalyptus	EL2	HL	0.16
Uroxys_sp1	6	Eucalyptus	EL2	HW	0.2
Uroxys_sp1	6	Eucalyptus	EL2	PL	0.2
Uroxys_sp1	6	Eucalyptus	EL2	PW	0.32

Uroxys_sp1	6	Eucalyptus	EL2	PH	0.2
Uroxys_sp1	6	Eucalyptus	EL2	EL	0.27
Uroxys_sp1	6	Eucalyptus	EL2	pTL	0.2
Uroxys_sp1	6	Eucalyptus	EL2	pTW	0.03
Uroxys_sp1	6	Eucalyptus	EL2	mTL	0.11
Uroxys_sp1	6	Eucalyptus	EL2	Biomass	0.00009
Uroxys_sp1	7	Eucalyptus	EL2	HL	0.15
Uroxys_sp1	7	Eucalyptus	EL2	HW	0.2
Uroxys_sp1	7	Eucalyptus	EL2	PL	0.26
Uroxys_sp1	7	Eucalyptus	EL2	PW	0.34
Uroxys_sp1	7	Eucalyptus	EL2	PH	0.18
Uroxys_sp1	7	Eucalyptus	EL2	EL	0.3
Uroxys_sp1	7	Eucalyptus	EL2	pTL	0.2
Uroxys_sp1	7	Eucalyptus	EL2	pTW	0.05
Uroxys_sp1	7	Eucalyptus	EL2	mTL	0.11
Uroxys_sp1	7	Eucalyptus	EL2	Biomass	0.00012
Uroxys_sp1	8	Eucalyptus	EL2	HL	0.18
Uroxys_sp1	8	Eucalyptus	EL2	HW	0.27
Uroxys_sp1	8	Eucalyptus	EL2	PL	0.28
Uroxys_sp1	8	Eucalyptus	EL2	PW	0.42
Uroxys_sp1	8	Eucalyptus	EL2	PH	0.28
Uroxys_sp1	8	Eucalyptus	EL2	EL	0.45
Uroxys_sp1	8	Eucalyptus	EL2	pTL	0.19
Uroxys_sp1	8	Eucalyptus	EL2	pTW	0.06
Uroxys_sp1	8	Eucalyptus	EL2	mTL	0.15
Uroxys_sp1	8	Eucalyptus	EL2	Biomass	0.00023
Uroxys_sp2	1	Forest	FL2	HL	0.13
Uroxys_sp2	1	Forest	FL2	HW	0.2
Uroxys_sp2	1	Forest	FL2	PL	0.18
Uroxys_sp2	1	Forest	FL2	PW	0.31
Uroxys_sp2	1	Forest	FL2	PH	0.13
Uroxys_sp2	1	Forest	FL2	EL	0.31
Uroxys_sp2	1	Forest	FL2	pTL	0.2
Uroxys_sp2	1	Forest	FL2	pTW	0.07
Uroxys_sp2	1	Forest	FL2	mTL	0.15
Uroxys_sp2	1	Forest	FL2	Biomass	0.00008
Uroxys_sp2	2	Forest	FL2	HL	0.14
Uroxys_sp2	2	Forest	FL2	HW	0.2
Uroxys_sp2	2	Forest	FL2	PL	0.18
Uroxys_sp2	2	Forest	FL2	PW	0.3
Uroxys_sp2	2	Forest	FL2	PH	0.13
Uroxys_sp2	2	Forest	FL2	EL	0.31
Uroxys_sp2	2	Forest	FL2	pTL	0.14

Uroxys_sp2	2	Forest	FL2	pTW	0.07
Uroxys_sp2	2	Forest	FL2	mTL	0.16
Uroxys_sp2	2	Forest	FL2	Biomass	0.00009
Uroxys_sp2	3	Forest	FL2	HL	0.13
Uroxys_sp2	3	Forest	FL2	HW	0.19
Uroxys_sp2	3	Forest	FL2	PL	0.17
Uroxys_sp2	3	Forest	FL2	PW	0.3
Uroxys_sp2	3	Forest	FL2	PH	0.13
Uroxys_sp2	3	Forest	FL2	EL	0.35
Uroxys_sp2	3	Forest	FL2	pTL	0.15
Uroxys_sp2	3	Forest	FL2	pTW	0.07
Uroxys_sp2	3	Forest	FL2	mTL	0.17
Uroxys_sp2	3	Forest	FL2	Biomass	0.00009
Uroxys_sp2	4	Forest	FL2	HL	0.15
Uroxys_sp2	4	Forest	FL2	HW	0.2
Uroxys_sp2	4	Forest	FL2	PL	0.16
Uroxys_sp2	4	Forest	FL2	PW	0.3
Uroxys_sp2	4	Forest	FL2	PH	0.13
Uroxys_sp2	4	Forest	FL2	EL	0.35
Uroxys_sp2	4	Forest	FL2	pTL	0.15
Uroxys_sp2	4	Forest	FL2	pTW	0.07
Uroxys_sp2	4	Forest	FL2	mTL	0.17
Uroxys_sp2	4	Forest	FL2	Biomass	0.0001
Uroxys_sp2	5	Forest	FL2	HL	0.16
Uroxys_sp2	5	Forest	FL2	HW	0.26
Uroxys_sp2	5	Forest	FL2	PL	0.23
Uroxys_sp2	5	Forest	FL2	PW	0.4
Uroxys_sp2	5	Forest	FL2	PH	0.16
Uroxys_sp2	5	Forest	FL2	EL	0.5
Uroxys_sp2	5	Forest	FL2	pTL	0.17
Uroxys_sp2	5	Forest	FL2	pTW	0.05
Uroxys_sp2	5	Forest	FL2	mTL	0.19
Uroxys_sp2	5	Forest	FL2	Biomass	0.00022
Uroxys_sp2	6	Forest	FL2	HL	0.17
Uroxys_sp2	6	Forest	FL2	HW	0.2
Uroxys_sp2	6	Forest	FL2	PL	0.16
Uroxys_sp2	6	Forest	FL2	PW	0.3
Uroxys_sp2	6	Forest	FL2	PH	0.13
Uroxys_sp2	6	Forest	FL2	EL	0.35
Uroxys_sp2	6	Forest	FL2	pTL	0.15
Uroxys_sp2	6	Forest	FL2	pTW	0.07
Uroxys_sp2	6	Forest	FL2	mTL	0.17
Uroxys_sp2	6	Forest	FL2	Biomass	0.00011

Uroxys_sp2	7	Forest	FL2	HL	0.16
Uroxys_sp2	7	Forest	FL2	HW	0.21
Uroxys_sp2	7	Forest	FL2	PL	0.17
Uroxys_sp2	7	Forest	FL2	PW	0.31
Uroxys_sp2	7	Forest	FL2	PH	0.14
Uroxys_sp2	7	Forest	FL2	EL	0.36
Uroxys_sp2	7	Forest	FL2	pTL	0.16
Uroxys_sp2	7	Forest	FL2	pTW	0.07
Uroxys_sp2	7	Forest	FL2	mTL	0.17
Uroxys_sp2	7	Forest	FL2	Biomass	0.00011
Uroxys_sp2	8	Forest	FL2	HL	0.13
Uroxys_sp2	8	Forest	FL2	HW	0.2
Uroxys_sp2	8	Forest	FL2	PL	0.18
Uroxys_sp2	8	Forest	FL2	PW	0.31
Uroxys_sp2	8	Forest	FL2	PH	0.13
Uroxys_sp2	8	Forest	FL2	EL	0.32
Uroxys_sp2	8	Forest	FL2	pTL	0.18
Uroxys_sp2	8	Forest	FL2	pTW	0.07
Uroxys_sp2	8	Forest	FL2	mTL	0.19
Uroxys_sp2	8	Forest	FL2	Biomass	0.00009
Uroxys_sp2	9	Forest	FL2	HL	0.16
Uroxys_sp2	9	Forest	FL2	HW	0.21
Uroxys_sp2	9	Forest	FL2	PL	0.18
Uroxys_sp2	9	Forest	FL2	PW	0.32
Uroxys_sp2	9	Forest	FL2	PH	0.13
Uroxys_sp2	9	Forest	FL2	EL	0.31
Uroxys_sp2	9	Forest	FL2	pTL	0.17
Uroxys_sp2	9	Forest	FL2	pTW	0.07
Uroxys_sp2	9	Forest	FL2	mTL	0.18
Uroxys_sp2	9	Forest	FL2	Biomass	0.00009
Uroxys_sp2	10	Forest	FL2	HL	0.14
Uroxys_sp2	10	Forest	FL2	HW	0.2
Uroxys_sp2	10	Forest	FL2	PL	0.18
Uroxys_sp2	10	Forest	FL2	PW	0.32
Uroxys_sp2	10	Forest	FL2	PH	0.13
Uroxys_sp2	10	Forest	FL2	EL	0.31
Uroxys_sp2	10	Forest	FL2	pTL	0.14
Uroxys_sp2	10	Forest	FL2	pTW	0.07
Uroxys_sp2	10	Forest	FL2	mTL	0.16
Uroxys_sp2	10	Forest	FL2	Biomass	0.00009
Uroxys_sp2	1	Forest	FL1	HL	0.12
Uroxys_sp2	1	Forest	FL1	HW	0.2
Uroxys_sp2	1	Forest	FL1	PL	0.18

Uroxys_sp2	1	Forest	FL1	PW	0.32
Uroxys_sp2	1	Forest	FL1	PH	0.13
Uroxys_sp2	1	Forest	FL1	EL	0.31
Uroxys_sp2	1	Forest	FL1	pTL	0.2
Uroxys_sp2	1	Forest	FL1	pTW	0.07
Uroxys_sp2	1	Forest	FL1	mTL	0.15
Uroxys_sp2	1	Forest	FL1	Biomass	0.00008
Uroxys_sp2	2	Forest	FL1	HL	0.14
Uroxys_sp2	2	Forest	FL1	HW	0.23
Uroxys_sp2	2	Forest	FL1	PL	0.2
Uroxys_sp2	2	Forest	FL1	PW	0.34
Uroxys_sp2	2	Forest	FL1	PH	0.15
Uroxys_sp2	2	Forest	FL1	EL	0.34
Uroxys_sp2	2	Forest	FL1	pTL	0.22
Uroxys_sp2	2	Forest	FL1	pTW	0.07
Uroxys_sp2	2	Forest	FL1	mTL	0.2
Uroxys_sp2	2	Forest	FL1	Biomass	0.00011
Uroxys_sp2	3	Forest	FL1	HL	0.13
Uroxys_sp2	3	Forest	FL1	HW	0.22
Uroxys_sp2	3	Forest	FL1	PL	0.21
Uroxys_sp2	3	Forest	FL1	PW	0.32
Uroxys_sp2	3	Forest	FL1	PH	0.14
Uroxys_sp2	3	Forest	FL1	EL	0.35
Uroxys_sp2	3	Forest	FL1	pTL	0.23
Uroxys_sp2	3	Forest	FL1	pTW	0.07
Uroxys_sp2	3	Forest	FL1	mTL	0.23
Uroxys_sp2	3	Forest	FL1	Biomass	0.00011
Uroxys_sp2	4	Forest	FL1	HL	0.11
Uroxys_sp2	4	Forest	FL1	HW	0.18
Uroxys_sp2	4	Forest	FL1	PL	0.16
Uroxys_sp2	4	Forest	FL1	PW	0.3
Uroxys_sp2	4	Forest	FL1	PH	0.12
Uroxys_sp2	4	Forest	FL1	EL	0.3
Uroxys_sp2	4	Forest	FL1	pTL	0.15
Uroxys_sp2	4	Forest	FL1	pTW	0.07
Uroxys_sp2	4	Forest	FL1	mTL	0.16
Uroxys_sp2	4	Forest	FL1	Biomass	0.00006
Uroxys_sp2	5	Forest	FL1	HL	0.12
Uroxys_sp2	5	Forest	FL1	HW	0.2
Uroxys_sp2	5	Forest	FL1	PL	0.18
Uroxys_sp2	5	Forest	FL1	PW	0.32
Uroxys_sp2	5	Forest	FL1	PH	0.13
Uroxys_sp2	5	Forest	FL1	EL	0.31

Uroxys_sp2	5	Forest	FL1	pTL	0.2
Uroxys_sp2	5	Forest	FL1	pTW	0.07
Uroxys_sp2	5	Forest	FL1	mTL	0.15
Uroxys_sp2	5	Forest	FL1	Biomass	0.00008
Uroxys_sp2	6	Forest	FL1	HL	0.14
Uroxys_sp2	6	Forest	FL1	HW	0.18
Uroxys_sp2	6	Forest	FL1	PL	0.15
Uroxys_sp2	6	Forest	FL1	PW	0.3
Uroxys_sp2	6	Forest	FL1	PH	0.12
Uroxys_sp2	6	Forest	FL1	EL	0.29
Uroxys_sp2	6	Forest	FL1	pTL	0.14
Uroxys_sp2	6	Forest	FL1	pTW	0.07
Uroxys_sp2	6	Forest	FL1	mTL	0.14
Uroxys_sp2	6	Forest	FL1	Biomass	0.00007
Uroxys_sp2	7	Forest	FL1	HL	0.14
Uroxys_sp2	7	Forest	FL1	HW	0.23
Uroxys_sp2	7	Forest	FL1	PL	0.2
Uroxys_sp2	7	Forest	FL1	PW	0.34
Uroxys_sp2	7	Forest	FL1	PH	0.15
Uroxys_sp2	7	Forest	FL1	EL	0.34
Uroxys_sp2	7	Forest	FL1	pTL	0.22
Uroxys_sp2	7	Forest	FL1	pTW	0.07
Uroxys_sp2	7	Forest	FL1	mTL	0.2
Uroxys_sp2	7	Forest	FL1	Biomass	0.00011
Uroxys_sp2	8	Forest	FL1	HL	0.18
Uroxys_sp2	8	Forest	FL1	HW	0.2
Uroxys_sp2	8	Forest	FL1	PL	0.2
Uroxys_sp2	8	Forest	FL1	PW	0.34
Uroxys_sp2	8	Forest	FL1	PH	0.15
Uroxys_sp2	8	Forest	FL1	EL	0.33
Uroxys_sp2	8	Forest	FL1	pTL	0.15
Uroxys_sp2	8	Forest	FL1	pTW	0.07
Uroxys_sp2	8	Forest	FL1	mTL	0.17
Uroxys_sp2	8	Forest	FL1	Biomass	0.00012
Uroxys_sp2	9	Forest	FL1	HL	0.16
Uroxys_sp2	9	Forest	FL1	HW	0.19
Uroxys_sp2	9	Forest	FL1	PL	0.21
Uroxys_sp2	9	Forest	FL1	PW	0.35
Uroxys_sp2	9	Forest	FL1	PH	0.16
Uroxys_sp2	9	Forest	FL1	EL	0.36
Uroxys_sp2	9	Forest	FL1	pTL	0.15
Uroxys_sp2	9	Forest	FL1	pTW	0.07
Uroxys_sp2	9	Forest	FL1	mTL	0.17

Uroxys_sp2	9	Forest	FL1	Biomass	0.00013
Uroxys_sp2	10	Forest	FL1	HL	0.13
Uroxys_sp2	10	Forest	FL1	HW	0.17
Uroxys_sp2	10	Forest	FL1	PL	0.19
Uroxys_sp2	10	Forest	FL1	PW	0.29
Uroxys_sp2	10	Forest	FL1	PH	0.18
Uroxys_sp2	10	Forest	FL1	EL	0.3
Uroxys_sp2	10	Forest	FL1	pTL	0.13
Uroxys_sp2	10	Forest	FL1	pTW	0.05
Uroxys_sp2	10	Forest	FL1	mTL	0.14
Uroxys_sp2	10	Forest	FL1	Biomass	0.00008
Uroxys_sp2	1	Pinus	PL1	HL	0.12
Uroxys_sp2	1	Pinus	PL1	HW	0.2
Uroxys_sp2	1	Pinus	PL1	PL	0.18
Uroxys_sp2	1	Pinus	PL1	PW	0.37
Uroxys_sp2	1	Pinus	PL1	PH	0.17
Uroxys_sp2	1	Pinus	PL1	EL	0.4
Uroxys_sp2	1	Pinus	PL1	pTL	0.13
Uroxys_sp2	1	Pinus	PL1	pTW	0.05
Uroxys_sp2	1	Pinus	PL1	mTL	0.18
Uroxys_sp2	1	Pinus	PL1	Biomass	0.00011
Uroxys_sp2	2	Pinus	PL1	HL	0.12
Uroxys_sp2	2	Pinus	PL1	HW	0.16
Uroxys_sp2	2	Pinus	PL1	PL	0.17
Uroxys_sp2	2	Pinus	PL1	PW	0.28
Uroxys_sp2	2	Pinus	PL1	PH	0.17
Uroxys_sp2	2	Pinus	PL1	EL	0.3
Uroxys_sp2	2	Pinus	PL1	pTL	0.13
Uroxys_sp2	2	Pinus	PL1	pTW	0.05
Uroxys_sp2	2	Pinus	PL1	mTL	0.12
Uroxys_sp2	2	Pinus	PL1	Biomass	0.00007
Uroxys_sp2	3	Pinus	PL1	HL	0.12
Uroxys_sp2	3	Pinus	PL1	HW	0.23
Uroxys_sp2	3	Pinus	PL1	PL	0.21
Uroxys_sp2	3	Pinus	PL1	PW	0.35
Uroxys_sp2	3	Pinus	PL1	PH	0.12
Uroxys_sp2	3	Pinus	PL1	EL	0.42
Uroxys_sp2	3	Pinus	PL1	pTL	0.12
Uroxys_sp2	3	Pinus	PL1	pTW	0.05
Uroxys_sp2	3	Pinus	PL1	mTL	0.17
Uroxys_sp2	3	Pinus	PL1	Biomass	0.00014
Uroxys_sp2	4	Pinus	PL1	HL	0.12
Uroxys_sp2	4	Pinus	PL1	HW	0.19

Uroxys_sp2	4	Pinus	PL1	PL	0.23
Uroxys_sp2	4	Pinus	PL1	PW	0.38
Uroxys_sp2	4	Pinus	PL1	PH	0.15
Uroxys_sp2	4	Pinus	PL1	EL	0.36
Uroxys_sp2	4	Pinus	PL1	pTL	0.19
Uroxys_sp2	4	Pinus	PL1	pTW	0.07
Uroxys_sp2	4	Pinus	PL1	mTL	0.17
Uroxys_sp2	4	Pinus	PL1	Biomass	0.00012
Uroxys_sp2	5	Pinus	PL1	HL	0.12
Uroxys_sp2	5	Pinus	PL1	HW	0.2
Uroxys_sp2	5	Pinus	PL1	PL	0.18
Uroxys_sp2	5	Pinus	PL1	PW	0.33
Uroxys_sp2	5	Pinus	PL1	PH	0.14
Uroxys_sp2	5	Pinus	PL1	EL	0.33
Uroxys_sp2	5	Pinus	PL1	pTL	0.15
Uroxys_sp2	5	Pinus	PL1	pTW	0.06
Uroxys_sp2	5	Pinus	PL1	mTL	0.15
Uroxys_sp2	5	Pinus	PL1	Biomass	0.00014
Uroxys_sp2	6	Pinus	PL1	HL	0.11
Uroxys_sp2	6	Pinus	PL1	HW	0.19
Uroxys_sp2	6	Pinus	PL1	PL	0.33
Uroxys_sp2	6	Pinus	PL1	PW	0.29
Uroxys_sp2	6	Pinus	PL1	PH	0.16
Uroxys_sp2	6	Pinus	PL1	EL	0.31
Uroxys_sp2	6	Pinus	PL1	pTL	0.15
Uroxys_sp2	6	Pinus	PL1	pTW	0.05
Uroxys_sp2	6	Pinus	PL1	mTL	0.15
Uroxys_sp2	6	Pinus	PL1	Biomass	0.00014
Uroxys_sp2	7	Pinus	PL1	HL	0.16
Uroxys_sp2	7	Pinus	PL1	HW	0.25
Uroxys_sp2	7	Pinus	PL1	PL	0.23
Uroxys_sp2	7	Pinus	PL1	PW	0.4
Uroxys_sp2	7	Pinus	PL1	PH	0.16
Uroxys_sp2	7	Pinus	PL1	EL	0.5
Uroxys_sp2	7	Pinus	PL1	pTL	0.17
Uroxys_sp2	7	Pinus	PL1	pTW	0.05
Uroxys_sp2	7	Pinus	PL1	mTL	0.19
Uroxys_sp2	7	Pinus	PL1	Biomass	0.00022
Uroxys_sp2	8	Pinus	PL1	HL	0.17
Uroxys_sp2	8	Pinus	PL1	HW	0.26
Uroxys_sp2	8	Pinus	PL1	PL	0.23
Uroxys_sp2	8	Pinus	PL1	PW	0.42
Uroxys_sp2	8	Pinus	PL1	PH	0.15

Uroxys_sp2	8	Pinus	PL1	EL	0.49
Uroxys_sp2	8	Pinus	PL1	pTL	0.23
Uroxys_sp2	8	Pinus	PL1	pTW	0.06
Uroxys_sp2	8	Pinus	PL1	mTL	0.2
Uroxys_sp2	8	Pinus	PL1	Biomass	0.00022
Uroxys_sp2	9	Pinus	PL1	HL	0.17
Uroxys_sp2	9	Pinus	PL1	HW	0.27
Uroxys_sp2	9	Pinus	PL1	PL	0.22
Uroxys_sp2	9	Pinus	PL1	PW	0.42
Uroxys_sp2	9	Pinus	PL1	PH	0.15
Uroxys_sp2	9	Pinus	PL1	EL	0.48
Uroxys_sp2	9	Pinus	PL1	pTL	0.26
Uroxys_sp2	9	Pinus	PL1	pTW	0.06
Uroxys_sp2	9	Pinus	PL1	mTL	0.19
Uroxys_sp2	9	Pinus	PL1	Biomass	0.00021
Uroxys_sp2	10	Pinus	PL1	HL	0.19
Uroxys_sp2	10	Pinus	PL1	HW	0.25
Uroxys_sp2	10	Pinus	PL1	PL	0.24
Uroxys_sp2	10	Pinus	PL1	PW	0.44
Uroxys_sp2	10	Pinus	PL1	PH	0.16
Uroxys_sp2	10	Pinus	PL1	EL	0.5
Uroxys_sp2	10	Pinus	PL1	pTL	0.25
Uroxys_sp2	10	Pinus	PL1	pTW	0.05
Uroxys_sp2	10	Pinus	PL1	mTL	0.2
Uroxys_sp2	10	Pinus	PL1	Biomass	0.00025
Uroxys_sp2	1	Pinus	PL2	HL	0.14
Uroxys_sp2	1	Pinus	PL2	HW	0.19
Uroxys_sp2	1	Pinus	PL2	PL	0.17
Uroxys_sp2	1	Pinus	PL2	PW	0.4
Uroxys_sp2	1	Pinus	PL2	PH	0.18
Uroxys_sp2	1	Pinus	PL2	EL	0.42
Uroxys_sp2	1	Pinus	PL2	pTL	0.15
Uroxys_sp2	1	Pinus	PL2	pTW	0.06
Uroxys_sp2	1	Pinus	PL2	mTL	0.17
Uroxys_sp2	1	Pinus	PL2	Biomass	0.00013
Uroxys_sp2	2	Pinus	PL2	HL	0.16
Uroxys_sp2	2	Pinus	PL2	HW	0.18
Uroxys_sp2	2	Pinus	PL2	PL	0.19
Uroxys_sp2	2	Pinus	PL2	PW	0.29
Uroxys_sp2	2	Pinus	PL2	PH	0.18
Uroxys_sp2	2	Pinus	PL2	EL	0.31
Uroxys_sp2	2	Pinus	PL2	pTL	0.11
Uroxys_sp2	2	Pinus	PL2	pTW	0.05

Uroxys_sp2	2	Pinus	PL2	mTL	0.12
Uroxys_sp2	2	Pinus	PL2	Biomass	0.0001
Uroxys_sp2	3	Pinus	PL2	HL	0.14
Uroxys_sp2	3	Pinus	PL2	HW	0.22
Uroxys_sp2	3	Pinus	PL2	PL	0.2
Uroxys_sp2	3	Pinus	PL2	PW	0.33
Uroxys_sp2	3	Pinus	PL2	PH	0.13
Uroxys_sp2	3	Pinus	PL2	EL	0.4
Uroxys_sp2	3	Pinus	PL2	pTL	0.12
Uroxys_sp2	3	Pinus	PL2	pTW	0.05
Uroxys_sp2	3	Pinus	PL2	mTL	0.14
Uroxys_sp2	3	Pinus	PL2	Biomass	0.00013
Uroxys_sp2	4	Pinus	PL2	HL	0.15
Uroxys_sp2	4	Pinus	PL2	HW	0.2
Uroxys_sp2	4	Pinus	PL2	PL	0.24
Uroxys_sp2	4	Pinus	PL2	PW	0.39
Uroxys_sp2	4	Pinus	PL2	PH	0.16
Uroxys_sp2	4	Pinus	PL2	EL	0.37
Uroxys_sp2	4	Pinus	PL2	pTL	0.16
Uroxys_sp2	4	Pinus	PL2	pTW	0.07
Uroxys_sp2	4	Pinus	PL2	mTL	0.17
Uroxys_sp2	4	Pinus	PL2	Biomass	0.00014
Uroxys_sp2	5	Pinus	PL2	HL	0.12
Uroxys_sp2	5	Pinus	PL2	HW	0.2
Uroxys_sp2	5	Pinus	PL2	PL	0.18
Uroxys_sp2	5	Pinus	PL2	PW	0.33
Uroxys_sp2	5	Pinus	PL2	PH	0.14
Uroxys_sp2	5	Pinus	PL2	EL	0.33
Uroxys_sp2	5	Pinus	PL2	pTL	0.15
Uroxys_sp2	5	Pinus	PL2	pTW	0.06
Uroxys_sp2	5	Pinus	PL2	mTL	0.15
Uroxys_sp2	5	Pinus	PL2	Biomass	0.00009
Uroxys_sp2	6	Pinus	PL2	HL	0.13
Uroxys_sp2	6	Pinus	PL2	HW	0.18
Uroxys_sp2	6	Pinus	PL2	PL	0.34
Uroxys_sp2	6	Pinus	PL2	PW	0.28
Uroxys_sp2	6	Pinus	PL2	PH	0.15
Uroxys_sp2	6	Pinus	PL2	EL	0.3
Uroxys_sp2	6	Pinus	PL2	pTL	0.14
Uroxys_sp2	6	Pinus	PL2	pTW	0.05
Uroxys_sp2	6	Pinus	PL2	mTL	0.15
Uroxys_sp2	6	Pinus	PL2	Biomass	0.00015
Uroxys_sp2	7	Pinus	PL2	HL	0.15

Uroxys_sp2	7	Pinus	PL2	HW	0.23
Uroxys_sp2	7	Pinus	PL2	PL	0.24
Uroxys_sp2	7	Pinus	PL2	PW	0.42
Uroxys_sp2	7	Pinus	PL2	PH	0.16
Uroxys_sp2	7	Pinus	PL2	EL	0.5
Uroxys_sp2	7	Pinus	PL2	pTL	0.17
Uroxys_sp2	7	Pinus	PL2	pTW	0.05
Uroxys_sp2	7	Pinus	PL2	mTL	0.17
Uroxys_sp2	7	Pinus	PL2	Biomass	0.00022
Uroxys_sp2	8	Pinus	PL2	HL	0.17
Uroxys_sp2	8	Pinus	PL2	HW	0.26
Uroxys_sp2	8	Pinus	PL2	PL	0.23
Uroxys_sp2	8	Pinus	PL2	PW	0.42
Uroxys_sp2	8	Pinus	PL2	PH	0.15
Uroxys_sp2	8	Pinus	PL2	EL	0.49
Uroxys_sp2	8	Pinus	PL2	pTL	0.19
Uroxys_sp2	8	Pinus	PL2	pTW	0.06
Uroxys_sp2	8	Pinus	PL2	mTL	0.2
Uroxys_sp2	8	Pinus	PL2	Biomass	0.00022
Uroxys_sp2	9	Pinus	PL2	HL	0.18
Uroxys_sp2	9	Pinus	PL2	HW	0.27
Uroxys_sp2	9	Pinus	PL2	PL	0.22
Uroxys_sp2	9	Pinus	PL2	PW	0.42
Uroxys_sp2	9	Pinus	PL2	PH	0.15
Uroxys_sp2	9	Pinus	PL2	EL	0.5
Uroxys_sp2	9	Pinus	PL2	pTL	0.26
Uroxys_sp2	9	Pinus	PL2	pTW	0.06
Uroxys_sp2	9	Pinus	PL2	mTL	0.19
Uroxys_sp2	9	Pinus	PL2	Biomass	0.00023
Uroxys_sp2	10	Pinus	PL2	HL	0.16
Uroxys_sp2	10	Pinus	PL2	HW	0.24
Uroxys_sp2	10	Pinus	PL2	PL	0.24
Uroxys_sp2	10	Pinus	PL2	PW	0.43
Uroxys_sp2	10	Pinus	PL2	PH	0.16
Uroxys_sp2	10	Pinus	PL2	EL	0.51
Uroxys_sp2	10	Pinus	PL2	pTL	0.21
Uroxys_sp2	10	Pinus	PL2	pTW	0.05
Uroxys_sp2	10	Pinus	PL2	mTL	0.2
Uroxys_sp2	10	Pinus	PL2	Biomass	0.00023
Uroxys_sp2	1	Eucalyptus	EL2	HL	0.18
Uroxys_sp2	1	Eucalyptus	EL2	HW	0.23
Uroxys_sp2	1	Eucalyptus	EL2	PL	0.22
Uroxys_sp2	1	Eucalyptus	EL2	PW	0.4

Uroxys_sp2	1	Eucalyptus	EL2	PH	0.15
Uroxys_sp2	1	Eucalyptus	EL2	EL	0.36
Uroxys_sp2	1	Eucalyptus	EL2	pTL	0.18
Uroxys_sp2	1	Eucalyptus	EL2	pTW	0.06
Uroxys_sp2	1	Eucalyptus	EL2	mTL	0.17
Uroxys_sp2	1	Eucalyptus	EL2	Biomass	0.00014
Uroxys_sp2	2	Eucalyptus	EL2	HL	0.12
Uroxys_sp2	2	Eucalyptus	EL2	HW	0.16
Uroxys_sp2	2	Eucalyptus	EL2	PL	0.17
Uroxys_sp2	2	Eucalyptus	EL2	PW	0.28
Uroxys_sp2	2	Eucalyptus	EL2	PH	0.16
Uroxys_sp2	2	Eucalyptus	EL2	EL	0.3
Uroxys_sp2	2	Eucalyptus	EL2	pTL	0.13
Uroxys_sp2	2	Eucalyptus	EL2	pTW	0.05
Uroxys_sp2	2	Eucalyptus	EL2	mTL	0.12
Uroxys_sp2	2	Eucalyptus	EL2	Biomass	0.00007
Uroxys_sp2	3	Eucalyptus	EL2	HL	0.15
Uroxys_sp2	3	Eucalyptus	EL2	HW	0.2
Uroxys_sp2	3	Eucalyptus	EL2	PL	0.2
Uroxys_sp2	3	Eucalyptus	EL2	PW	0.32
Uroxys_sp2	3	Eucalyptus	EL2	PH	0.15
Uroxys_sp2	3	Eucalyptus	EL2	EL	0.36
Uroxys_sp2	3	Eucalyptus	EL2	pTL	0.14
Uroxys_sp2	3	Eucalyptus	EL2	pTW	0.06
Uroxys_sp2	3	Eucalyptus	EL2	mTL	0.15
Uroxys_sp2	3	Eucalyptus	EL2	Biomass	0.00012
Uroxys_sp2	4	Eucalyptus	EL2	HL	0.11
Uroxys_sp2	4	Eucalyptus	EL2	HW	0.16
Uroxys_sp2	4	Eucalyptus	EL2	PL	0.17
Uroxys_sp2	4	Eucalyptus	EL2	PW	0.27
Uroxys_sp2	4	Eucalyptus	EL2	PH	0.15
Uroxys_sp2	4	Eucalyptus	EL2	EL	0.27
Uroxys_sp2	4	Eucalyptus	EL2	pTL	0.13
Uroxys_sp2	4	Eucalyptus	EL2	pTW	0.05
Uroxys_sp2	4	Eucalyptus	EL2	mTL	0.12
Uroxys_sp2	4	Eucalyptus	EL2	Biomass	0.00006
Uroxys_sp2	5	Eucalyptus	EL2	HL	0.16
Uroxys_sp2	5	Eucalyptus	EL2	HW	0.22
Uroxys_sp2	5	Eucalyptus	EL2	PL	0.19
Uroxys_sp2	5	Eucalyptus	EL2	PW	0.3
Uroxys_sp2	5	Eucalyptus	EL2	PH	0.15
Uroxys_sp2	5	Eucalyptus	EL2	EL	0.4
Uroxys_sp2	5	Eucalyptus	EL2	pTL	0.14

Uroxys_sp2	5	Eucalyptus	EL2	pTW	0.06
Uroxys_sp2	5	Eucalyptus	EL2	mTL	0.15
Uroxys_sp2	5	Eucalyptus	EL2	Biomass	0.00014
Uroxys_sp2	6	Eucalyptus	EL2	HL	0.17
Uroxys_sp2	6	Eucalyptus	EL2	HW	0.22
Uroxys_sp2	6	Eucalyptus	EL2	PL	0.23
Uroxys_sp2	6	Eucalyptus	EL2	PW	0.34
Uroxys_sp2	6	Eucalyptus	EL2	PH	0.17
Uroxys_sp2	6	Eucalyptus	EL2	EL	0.33
Uroxys_sp2	6	Eucalyptus	EL2	pTL	0.15
Uroxys_sp2	6	Eucalyptus	EL2	pTW	0.06
Uroxys_sp2	6	Eucalyptus	EL2	mTL	0.16
Uroxys_sp2	6	Eucalyptus	EL2	Biomass	0.00013
Uroxys_sp2	7	Eucalyptus	EL2	HL	0.19
Uroxys_sp2	7	Eucalyptus	EL2	HW	0.23
Uroxys_sp2	7	Eucalyptus	EL2	PL	0.25
Uroxys_sp2	7	Eucalyptus	EL2	PW	0.36
Uroxys_sp2	7	Eucalyptus	EL2	PH	0.17
Uroxys_sp2	7	Eucalyptus	EL2	EL	0.35
Uroxys_sp2	7	Eucalyptus	EL2	pTL	0.16
Uroxys_sp2	7	Eucalyptus	EL2	pTW	0.06
Uroxys_sp2	7	Eucalyptus	EL2	mTL	0.18
Uroxys_sp2	7	Eucalyptus	EL2	Biomass	0.00016
Uroxys_sp2	8	Eucalyptus	EL2	HL	0.17
Uroxys_sp2	8	Eucalyptus	EL2	HW	0.22
Uroxys_sp2	8	Eucalyptus	EL2	PL	0.23
Uroxys_sp2	8	Eucalyptus	EL2	PW	0.34
Uroxys_sp2	8	Eucalyptus	EL2	PH	0.17
Uroxys_sp2	8	Eucalyptus	EL2	EL	0.33
Uroxys_sp2	8	Eucalyptus	EL2	pTL	0.15
Uroxys_sp2	8	Eucalyptus	EL2	pTW	0.06
Uroxys_sp2	8	Eucalyptus	EL2	mTL	0.16
Uroxys_sp2	8	Eucalyptus	EL2	Biomass	0.00013
Uroxys_sp2	9	Eucalyptus	EL2	HL	0.13
Uroxys_sp2	9	Eucalyptus	EL2	HW	0.16
Uroxys_sp2	9	Eucalyptus	EL2	PL	0.17
Uroxys_sp2	9	Eucalyptus	EL2	PW	0.3
Uroxys_sp2	9	Eucalyptus	EL2	PH	0.16
Uroxys_sp2	9	Eucalyptus	EL2	EL	0.3
Uroxys_sp2	9	Eucalyptus	EL2	pTL	0.12
Uroxys_sp2	9	Eucalyptus	EL2	pTW	0.05
Uroxys_sp2	9	Eucalyptus	EL2	mTL	0.14
Uroxys_sp2	9	Eucalyptus	EL2	Biomass	0.00007

Uroxys_sp2	10	Eucalyptus	EL2	HL	0.16
Uroxys_sp2	10	Eucalyptus	EL2	HW	0.18
Uroxys_sp2	10	Eucalyptus	EL2	PL	0.19
Uroxys_sp2	10	Eucalyptus	EL2	PW	0.3
Uroxys_sp2	10	Eucalyptus	EL2	PH	0.15
Uroxys_sp2	10	Eucalyptus	EL2	EL	0.32
Uroxys_sp2	10	Eucalyptus	EL2	pTL	0.14
Uroxys_sp2	10	Eucalyptus	EL2	pTW	0.06
Uroxys_sp2	10	Eucalyptus	EL2	mTL	0.14
Uroxys_sp2	10	Eucalyptus	EL2	Biomass	0.0001
Uroxys_sp2	1	Eucalyptus	EL1	HL	0.11
Uroxys_sp2	1	Eucalyptus	EL1	HW	0.16
Uroxys_sp2	1	Eucalyptus	EL1	PL	0.17
Uroxys_sp2	1	Eucalyptus	EL1	PW	0.28
Uroxys_sp2	1	Eucalyptus	EL1	PH	0.14
Uroxys_sp2	1	Eucalyptus	EL1	EL	0.31
Uroxys_sp2	1	Eucalyptus	EL1	pTL	0.12
Uroxys_sp2	1	Eucalyptus	EL1	pTW	0.06
Uroxys_sp2	1	Eucalyptus	EL1	mTL	0.14
Uroxys_sp2	1	Eucalyptus	EL1	Biomass	0.00007
Uroxys_sp2	2	Eucalyptus	EL1	HL	0.11
Uroxys_sp2	2	Eucalyptus	EL1	HW	0.15
Uroxys_sp2	2	Eucalyptus	EL1	PL	0.17
Uroxys_sp2	2	Eucalyptus	EL1	PW	0.3
Uroxys_sp2	2	Eucalyptus	EL1	PH	0.14
Uroxys_sp2	2	Eucalyptus	EL1	EL	0.33
Uroxys_sp2	2	Eucalyptus	EL1	pTL	0.13
Uroxys_sp2	2	Eucalyptus	EL1	pTW	0.06
Uroxys_sp2	2	Eucalyptus	EL1	mTL	0.16
Uroxys_sp2	2	Eucalyptus	EL1	Biomass	0.00008
Onoreidium_ohausi	1	Eucalyptus	ES1	HL	0.07
Onoreidium_ohausi	1	Eucalyptus	ES1	HW	0.15
Onoreidium_ohausi	1	Eucalyptus	ES1	PL	0.12
Onoreidium_ohausi	1	Eucalyptus	ES1	PW	0.22
Onoreidium_ohausi	1	Eucalyptus	ES1	PH	0.15
Onoreidium_ohausi	1	Eucalyptus	ES1	EL	0.30
Onoreidium_ohausi	1	Eucalyptus	ES1	pTL	0.10
Onoreidium_ohausi	1	Eucalyptus	ES1	pTW	0.04
Onoreidium_ohausi	1	Eucalyptus	ES1	mTL	0.12
Onoreidium_ohausi	1	Eucalyptus	ES1	Biomass	4.71
Dichotomius_cotopaxi	1	Pinus	PS1	HL	0.66
Dichotomius_cotopaxi	1	Pinus	PS1	HW	0.70
Dichotomius_cotopaxi	1	Pinus	PS1	PL	0.70

Dichotomius_cotopaxi	1	Pinus	PS1	PW	1.20
Dichotomius_cotopaxi	1	Pinus	PS1	PH	0.70
Dichotomius_cotopaxi	1	Pinus	PS1	EL	1.05
Dichotomius_cotopaxi	1	Pinus	PS1	pTL	0.45
Dichotomius_cotopaxi	1	Pinus	PS1	pTW	0.22
Dichotomius_cotopaxi	1	Pinus	PS1	mTL	0.45
Dichotomius_cotopaxi	1	Pinus	PS1	Biomass	0.0030
Dichotomius_cotopaxi	1	Eucalyptus	ES1	HL	0.65
Dichotomius_cotopaxi	1	Eucalyptus	ES1	HW	0.70
Dichotomius_cotopaxi	1	Eucalyptus	ES1	PL	0.75
Dichotomius_cotopaxi	1	Eucalyptus	ES1	PW	1.20
Dichotomius_cotopaxi	1	Eucalyptus	ES1	PH	0.80
Dichotomius_cotopaxi	1	Eucalyptus	ES1	EL	1.10
Dichotomius_cotopaxi	1	Eucalyptus	ES1	pTL	0.45
Dichotomius_cotopaxi	1	Eucalyptus	ES1	pTW	0.23
Dichotomius_cotopaxi	1	Eucalyptus	ES1	mTL	0.45
Dichotomius_cotopaxi	1	Eucalyptus	ES1	Biomass	0.0034
Homocopris_buckleyi	1	Forest	FS1	HL	0.45
Homocopris_buckleyi	1	Forest	FS1	HW	0.73
Homocopris_buckleyi	1	Forest	FS1	PL	0.60
Homocopris_buckleyi	1	Forest	FS1	PW	1.00
Homocopris_buckleyi	1	Forest	FS1	PH	0.75
Homocopris_buckleyi	1	Forest	FS1	EL	1.00
Homocopris_buckleyi	1	Forest	FS1	pTL	0.41
Homocopris_buckleyi	1	Forest	FS1	pTW	0.13
Homocopris_buckleyi	1	Forest	FS1	mTL	0.45
Homocopris_buckleyi	1	Forest	FS1	Biomass	0.0020
Homocopris_buckleyi	2	Forest	FS1	HL	0.45
Homocopris_buckleyi	2	Forest	FS1	HW	0.65
Homocopris_buckleyi	2	Forest	FS1	PL	0.70
Homocopris_buckleyi	2	Forest	FS1	PW	1.00
Homocopris_buckleyi	2	Forest	FS1	PH	0.73
Homocopris_buckleyi	2	Forest	FS1	EL	0.94
Homocopris_buckleyi	2	Forest	FS1	pTL	0.38
Homocopris_buckleyi	2	Forest	FS1	pTW	0.15
Homocopris_buckleyi	2	Forest	FS1	mTL	0.43
Homocopris_buckleyi	2	Forest	FS1	Biomass	0.0021
Homocopris_buckleyi	3	Forest	FS1	HL	0.45
Homocopris_buckleyi	3	Forest	FS1	HW	0.67
Homocopris_buckleyi	3	Forest	FS1	PL	0.75
Homocopris_buckleyi	3	Forest	FS1	PW	1.04
Homocopris_buckleyi	3	Forest	FS1	PH	0.75
Homocopris_buckleyi	3	Forest	FS1	EL	0.95

Homocopris_buckleyi	3	Forest	FS1	pTL	0.39
Homocopris_buckleyi	3	Forest	FS1	pTW	0.16
Homocopris_buckleyi	3	Forest	FS1	mTL	0.45
Homocopris_buckleyi	3	Forest	FS1	Biomass	0.0023
Homocopris_buckleyi	4	Forest	FS1	HL	0.50
Homocopris_buckleyi	4	Forest	FS1	HW	0.70
Homocopris_buckleyi	4	Forest	FS1	PL	0.75
Homocopris_buckleyi	4	Forest	FS1	PW	1.02
Homocopris_buckleyi	4	Forest	FS1	PH	0.70
Homocopris_buckleyi	4	Forest	FS1	EL	0.93
Homocopris_buckleyi	4	Forest	FS1	pTL	0.43
Homocopris_buckleyi	4	Forest	FS1	pTW	0.15
Homocopris_buckleyi	4	Forest	FS1	mTL	0.36
Homocopris_buckleyi	4	Forest	FS1	Biomass	0.0024
Homocopris_buckleyi	5	Forest	FS1	HL	0.50
Homocopris_buckleyi	5	Forest	FS1	HW	0.70
Homocopris_buckleyi	5	Forest	FS1	PL	0.65
Homocopris_buckleyi	5	Forest	FS1	PW	1.05
Homocopris_buckleyi	5	Forest	FS1	PH	0.60
Homocopris_buckleyi	5	Forest	FS1	EL	0.80
Homocopris_buckleyi	5	Forest	FS1	pTL	0.40
Homocopris_buckleyi	5	Forest	FS1	pTW	0.17
Homocopris_buckleyi	5	Forest	FS1	mTL	0.43
Homocopris_buckleyi	5	Forest	FS1	Biomass	0.0017
Homocopris_buckleyi	6	Forest	FS1	HL	0.47
Homocopris_buckleyi	6	Forest	FS1	HW	0.65
Homocopris_buckleyi	6	Forest	FS1	PL	0.60
Homocopris_buckleyi	6	Forest	FS1	PW	1.05
Homocopris_buckleyi	6	Forest	FS1	PH	0.60
Homocopris_buckleyi	6	Forest	FS1	EL	0.90
Homocopris_buckleyi	6	Forest	FS1	pTL	0.33
Homocopris_buckleyi	6	Forest	FS1	pTW	0.16
Homocopris_buckleyi	6	Forest	FS1	mTL	0.43
Homocopris_buckleyi	6	Forest	FS1	Biomass	0.0018
Homocopris_buckleyi	7	Forest	FS1	HL	0.50
Homocopris_buckleyi	7	Forest	FS1	HW	0.75
Homocopris_buckleyi	7	Forest	FS1	PL	0.75
Homocopris_buckleyi	7	Forest	FS1	PW	1.20
Homocopris_buckleyi	7	Forest	FS1	PH	0.60
Homocopris_buckleyi	7	Forest	FS1	EL	1.00
Homocopris_buckleyi	7	Forest	FS1	pTL	0.43
Homocopris_buckleyi	7	Forest	FS1	pTW	0.20
Homocopris_buckleyi	7	Forest	FS1	mTL	0.45

Homocopris_buckleyi	7	Forest	FS1	Biomass	0.0025
Homocopris_buckleyi	8	Forest	FS1	HL	0.40
Homocopris_buckleyi	8	Forest	FS1	HW	0.63
Homocopris_buckleyi	8	Forest	FS1	PL	0.63
Homocopris_buckleyi	8	Forest	FS1	PW	0.97
Homocopris_buckleyi	8	Forest	FS1	PH	0.65
Homocopris_buckleyi	8	Forest	FS1	EL	0.95
Homocopris_buckleyi	8	Forest	FS1	pTL	0.45
Homocopris_buckleyi	8	Forest	FS1	pTW	0.17
Homocopris_buckleyi	8	Forest	FS1	mTL	0.35
Homocopris_buckleyi	8	Forest	FS1	Biomass	0.0018
Homocopris_buckleyi	9	Forest	FS1	HL	0.45
Homocopris_buckleyi	9	Forest	FS1	HW	0.70
Homocopris_buckleyi	9	Forest	FS1	PL	0.70
Homocopris_buckleyi	9	Forest	FS1	PW	1.10
Homocopris_buckleyi	9	Forest	FS1	PH	0.60
Homocopris_buckleyi	9	Forest	FS1	EL	1.00
Homocopris_buckleyi	9	Forest	FS1	pTL	0.41
Homocopris_buckleyi	9	Forest	FS1	pTW	0.20
Homocopris_buckleyi	9	Forest	FS1	mTL	0.48
Homocopris_buckleyi	9	Forest	FS1	Biomass	0.0023
Homocopris_buckleyi	10	Forest	FS1	HL	0.50
Homocopris_buckleyi	10	Forest	FS1	HW	0.75
Homocopris_buckleyi	10	Forest	FS1	PL	0.71
Homocopris_buckleyi	10	Forest	FS1	PW	1.20
Homocopris_buckleyi	10	Forest	FS1	PH	0.60
Homocopris_buckleyi	10	Forest	FS1	EL	1.00
Homocopris_buckleyi	10	Forest	FS1	pTL	0.42
Homocopris_buckleyi	10	Forest	FS1	pTW	0.20
Homocopris_buckleyi	10	Forest	FS1	mTL	0.45
Homocopris_buckleyi	10	Forest	FS1	Biomass	0.0024
Homocopris_buckleyi	1	Pinus	PS2	HL	0.43
Homocopris_buckleyi	1	Pinus	PS2	HW	0.65
Homocopris_buckleyi	1	Pinus	PS2	PL	0.70
Homocopris_buckleyi	1	Pinus	PS2	PW	1.03
Homocopris_buckleyi	1	Pinus	PS2	PH	0.70
Homocopris_buckleyi	1	Pinus	PS2	EL	0.86
Homocopris_buckleyi	1	Pinus	PS2	pTL	0.33
Homocopris_buckleyi	1	Pinus	PS2	pTW	0.15
Homocopris_buckleyi	1	Pinus	PS2	mTL	0.48
Homocopris_buckleyi	1	Pinus	PS2	Biomass	0.0018
Homocopris_buckleyi	2	Pinus	PS2	HL	0.40
Homocopris_buckleyi	2	Pinus	PS2	HW	0.70

Homocopris_buckleyi	2	Pinus	PS2	PL	0.72
Homocopris_buckleyi	2	Pinus	PS2	PW	1.10
Homocopris_buckleyi	2	Pinus	PS2	PH	0.65
Homocopris_buckleyi	2	Pinus	PS2	EL	1.10
Homocopris_buckleyi	2	Pinus	PS2	pTL	0.50
Homocopris_buckleyi	2	Pinus	PS2	pTW	0.15
Homocopris_buckleyi	2	Pinus	PS2	mTL	0.40
Homocopris_buckleyi	2	Pinus	PS2	Biomass	0.0025
Homocopris_buckleyi	3	Pinus	PS2	HL	0.50
Homocopris_buckleyi	3	Pinus	PS2	HW	0.70
Homocopris_buckleyi	3	Pinus	PS2	PL	0.75
Homocopris_buckleyi	3	Pinus	PS2	PW	1.12
Homocopris_buckleyi	3	Pinus	PS2	PH	0.75
Homocopris_buckleyi	3	Pinus	PS2	EL	1.00
Homocopris_buckleyi	3	Pinus	PS2	pTL	0.40
Homocopris_buckleyi	3	Pinus	PS2	pTW	0.16
Homocopris_buckleyi	3	Pinus	PS2	mTL	0.50
Homocopris_buckleyi	3	Pinus	PS2	Biomass	0.0025
Homocopris_buckleyi	4	Pinus	PS2	HL	0.50
Homocopris_buckleyi	4	Pinus	PS2	HW	0.65
Homocopris_buckleyi	4	Pinus	PS2	PL	0.70
Homocopris_buckleyi	4	Pinus	PS2	PW	1.05
Homocopris_buckleyi	4	Pinus	PS2	PH	0.70
Homocopris_buckleyi	4	Pinus	PS2	EL	0.93
Homocopris_buckleyi	4	Pinus	PS2	pTL	0.35
Homocopris_buckleyi	4	Pinus	PS2	pTW	0.15
Homocopris_buckleyi	4	Pinus	PS2	mTL	0.46
Homocopris_buckleyi	4	Pinus	PS2	Biomass	0.0022
Homocopris_buckleyi	5	Pinus	PS2	HL	0.40
Homocopris_buckleyi	5	Pinus	PS2	HW	0.65
Homocopris_buckleyi	5	Pinus	PS2	PL	0.70
Homocopris_buckleyi	5	Pinus	PS2	PW	1.00
Homocopris_buckleyi	5	Pinus	PS2	PH	0.73
Homocopris_buckleyi	5	Pinus	PS2	EL	1.00
Homocopris_buckleyi	5	Pinus	PS2	pTL	0.35
Homocopris_buckleyi	5	Pinus	PS2	pTW	0.13
Homocopris_buckleyi	5	Pinus	PS2	mTL	0.45
Homocopris_buckleyi	5	Pinus	PS2	Biomass	0.0022
Homocopris_buckleyi	6	Pinus	PS2	HL	0.53
Homocopris_buckleyi	6	Pinus	PS2	HW	0.75
Homocopris_buckleyi	6	Pinus	PS2	PL	0.70
Homocopris_buckleyi	6	Pinus	PS2	PW	1.15
Homocopris_buckleyi	6	Pinus	PS2	PH	0.66

Homocopris_buckleyi	6	Pinus	PS2	EL	1.05
Homocopris_buckleyi	6	Pinus	PS2	pTL	0.34
Homocopris_buckleyi	6	Pinus	PS2	pTW	0.22
Homocopris_buckleyi	6	Pinus	PS2	mTL	0.50
Homocopris_buckleyi	6	Pinus	PS2	Biomass	0.0026
Homocopris_buckleyi	7	Pinus	PS2	HL	0.50
Homocopris_buckleyi	7	Pinus	PS2	HW	0.73
Homocopris_buckleyi	7	Pinus	PS2	PL	0.65
Homocopris_buckleyi	7	Pinus	PS2	PW	1.10
Homocopris_buckleyi	7	Pinus	PS2	PH	0.65
Homocopris_buckleyi	7	Pinus	PS2	EL	1.00
Homocopris_buckleyi	7	Pinus	PS2	pTL	0.37
Homocopris_buckleyi	7	Pinus	PS2	pTW	0.11
Homocopris_buckleyi	7	Pinus	PS2	mTL	0.47
Homocopris_buckleyi	7	Pinus	PS2	Biomass	0.0023
Homocopris_buckleyi	8	Pinus	PS2	HL	0.45
Homocopris_buckleyi	8	Pinus	PS2	HW	0.70
Homocopris_buckleyi	8	Pinus	PS2	PL	0.65
Homocopris_buckleyi	8	Pinus	PS2	PW	1.05
Homocopris_buckleyi	8	Pinus	PS2	PH	0.50
Homocopris_buckleyi	8	Pinus	PS2	EL	0.90
Homocopris_buckleyi	8	Pinus	PS2	pTL	0.40
Homocopris_buckleyi	8	Pinus	PS2	pTW	0.20
Homocopris_buckleyi	8	Pinus	PS2	mTL	0.45
Homocopris_buckleyi	8	Pinus	PS2	Biomass	0.0018
Homocopris_buckleyi	9	Pinus	PS2	HL	0.45
Homocopris_buckleyi	9	Pinus	PS2	HW	0.63
Homocopris_buckleyi	9	Pinus	PS2	PL	0.55
Homocopris_buckleyi	9	Pinus	PS2	PW	0.93
Homocopris_buckleyi	9	Pinus	PS2	PH	0.56
Homocopris_buckleyi	9	Pinus	PS2	EL	0.80
Homocopris_buckleyi	9	Pinus	PS2	pTL	0.32
Homocopris_buckleyi	9	Pinus	PS2	pTW	0.14
Homocopris_buckleyi	9	Pinus	PS2	mTL	0.40
Homocopris_buckleyi	9	Pinus	PS2	Biomass	0.0014
Homocopris_buckleyi	10	Pinus	PS2	HL	0.38
Homocopris_buckleyi	10	Pinus	PS2	HW	0.60
Homocopris_buckleyi	10	Pinus	PS2	PL	0.59
Homocopris_buckleyi	10	Pinus	PS2	PW	0.90
Homocopris_buckleyi	10	Pinus	PS2	PH	0.50
Homocopris_buckleyi	10	Pinus	PS2	EL	0.88
Homocopris_buckleyi	10	Pinus	PS2	pTL	0.30
Homocopris_buckleyi	10	Pinus	PS2	pTW	0.15

Homocopris_buckleyi	10	Pinus	PS2	mTL	0.40
Homocopris_buckleyi	10	Pinus	PS2	Biomass	0.0015
Homocopris_buckleyi	1	Forest	FS2	HL	0.40
Homocopris_buckleyi	1	Forest	FS2	HW	0.62
Homocopris_buckleyi	1	Forest	FS2	PL	0.63
Homocopris_buckleyi	1	Forest	FS2	PW	0.96
Homocopris_buckleyi	1	Forest	FS2	PH	0.60
Homocopris_buckleyi	1	Forest	FS2	EL	0.90
Homocopris_buckleyi	1	Forest	FS2	pTL	0.37
Homocopris_buckleyi	1	Forest	FS2	pTW	0.14
Homocopris_buckleyi	1	Forest	FS2	mTL	0.42
Homocopris_buckleyi	1	Forest	FS2	Biomass	0.0017
Homocopris_buckleyi	2	Forest	FS2	HL	0.40
Homocopris_buckleyi	2	Forest	FS2	HW	0.65
Homocopris_buckleyi	2	Forest	FS2	PL	0.67
Homocopris_buckleyi	2	Forest	FS2	PW	1.00
Homocopris_buckleyi	2	Forest	FS2	PH	0.65
Homocopris_buckleyi	2	Forest	FS2	EL	0.92
Homocopris_buckleyi	2	Forest	FS2	pTL	0.34
Homocopris_buckleyi	2	Forest	FS2	pTW	0.12
Homocopris_buckleyi	2	Forest	FS2	mTL	0.43
Homocopris_buckleyi	2	Forest	FS2	Biomass	0.0018
Homocopris_buckleyi	3	Forest	FS2	HL	0.45
Homocopris_buckleyi	3	Forest	FS2	HW	0.74
Homocopris_buckleyi	3	Forest	FS2	PL	0.75
Homocopris_buckleyi	3	Forest	FS2	PW	1.17
Homocopris_buckleyi	3	Forest	FS2	PH	0.70
Homocopris_buckleyi	3	Forest	FS2	EL	0.95
Homocopris_buckleyi	3	Forest	FS2	pTL	0.43
Homocopris_buckleyi	3	Forest	FS2	pTW	0.17
Homocopris_buckleyi	3	Forest	FS2	mTL	0.46
Homocopris_buckleyi	3	Forest	FS2	Biomass	0.0023
Homocopris_buckleyi	4	Forest	FS2	HL	0.46
Homocopris_buckleyi	4	Forest	FS2	HW	0.73
Homocopris_buckleyi	4	Forest	FS2	PL	0.72
Homocopris_buckleyi	4	Forest	FS2	PW	1.12
Homocopris_buckleyi	4	Forest	FS2	PH	0.65
Homocopris_buckleyi	4	Forest	FS2	EL	1.00
Homocopris_buckleyi	4	Forest	FS2	pTL	0.40
Homocopris_buckleyi	4	Forest	FS2	pTW	0.17
Homocopris_buckleyi	4	Forest	FS2	mTL	0.45
Homocopris_buckleyi	4	Forest	FS2	Biomass	0.0024
Homocopris_buckleyi	5	Forest	FS2	HL	0.45

Homocopris_buckleyi	5	Forest	FS2	HW	0.65
Homocopris_buckleyi	5	Forest	FS2	PL	0.63
Homocopris_buckleyi	5	Forest	FS2	PW	0.98
Homocopris_buckleyi	5	Forest	FS2	PH	0.70
Homocopris_buckleyi	5	Forest	FS2	EL	0.91
Homocopris_buckleyi	5	Forest	FS2	pTL	0.35
Homocopris_buckleyi	5	Forest	FS2	pTW	0.13
Homocopris_buckleyi	5	Forest	FS2	mTL	0.40
Homocopris_buckleyi	5	Forest	FS2	Biomass	0.0038
Homocopris_buckleyi	6	Forest	FS2	HL	0.50
Homocopris_buckleyi	6	Forest	FS2	HW	0.80
Homocopris_buckleyi	6	Forest	FS2	PL	0.75
Homocopris_buckleyi	6	Forest	FS2	PW	1.20
Homocopris_buckleyi	6	Forest	FS2	PH	0.52
Homocopris_buckleyi	6	Forest	FS2	EL	1.05
Homocopris_buckleyi	6	Forest	FS2	pTL	0.40
Homocopris_buckleyi	6	Forest	FS2	pTW	0.22
Homocopris_buckleyi	6	Forest	FS2	mTL	0.45
Homocopris_buckleyi	6	Forest	FS2	Biomass	0.0027
Homocopris_buckleyi	7	Forest	FS2	HL	0.45
Homocopris_buckleyi	7	Forest	FS2	HW	0.66
Homocopris_buckleyi	7	Forest	FS2	PL	0.65
Homocopris_buckleyi	7	Forest	FS2	PW	1.05
Homocopris_buckleyi	7	Forest	FS2	PH	0.60
Homocopris_buckleyi	7	Forest	FS2	EL	0.86
Homocopris_buckleyi	7	Forest	FS2	pTL	0.32
Homocopris_buckleyi	7	Forest	FS2	pTW	0.19
Homocopris_buckleyi	7	Forest	FS2	mTL	0.43
Homocopris_buckleyi	7	Forest	FS2	Biomass	0.0017
Homocopris_buckleyi	8	Forest	FS2	HL	0.50
Homocopris_buckleyi	8	Forest	FS2	HW	0.72
Homocopris_buckleyi	8	Forest	FS2	PL	0.70
Homocopris_buckleyi	8	Forest	FS2	PW	1.10
Homocopris_buckleyi	8	Forest	FS2	PH	0.58
Homocopris_buckleyi	8	Forest	FS2	EL	1.00
Homocopris_buckleyi	8	Forest	FS2	pTL	0.40
Homocopris_buckleyi	8	Forest	FS2	pTW	0.20
Homocopris_buckleyi	8	Forest	FS2	mTL	0.50
Homocopris_buckleyi	8	Forest	FS2	Biomass	0.0024
Homocopris_buckleyi	9	Forest	FS2	HL	0.45
Homocopris_buckleyi	9	Forest	FS2	HW	0.63
Homocopris_buckleyi	9	Forest	FS2	PL	0.58
Homocopris_buckleyi	9	Forest	FS2	PW	0.95

Homocopris_buckleyi	9	Forest	FS2	PH	0.65
Homocopris_buckleyi	9	Forest	FS2	EL	0.82
Homocopris_buckleyi	9	Forest	FS2	pTL	0.33
Homocopris_buckleyi	9	Forest	FS2	pTW	0.11
Homocopris_buckleyi	9	Forest	FS2	mTL	0.40
Homocopris_buckleyi	9	Forest	FS2	Biomass	0.0015
Homocopris_buckleyi	10	Forest	FS2	HL	0.50
Homocopris_buckleyi	10	Forest	FS2	HW	0.75
Homocopris_buckleyi	10	Forest	FS2	PL	0.75
Homocopris_buckleyi	10	Forest	FS2	PW	1.20
Homocopris_buckleyi	10	Forest	FS2	PH	0.70
Homocopris_buckleyi	10	Forest	FS2	EL	1.00
Homocopris_buckleyi	10	Forest	FS2	pTL	0.43
Homocopris_buckleyi	10	Forest	FS2	pTW	0.23
Homocopris_buckleyi	10	Forest	FS2	mTL	0.47
Homocopris_buckleyi	10	Forest	FS2	Biomass	0.0025
Onthophagus_curvicornis	1	Pinus	PS1	HL	0.30
Onthophagus_curvicornis	1	Pinus	PS1	HW	0.33
Onthophagus_curvicornis	1	Pinus	PS1	PL	0.30
Onthophagus_curvicornis	1	Pinus	PS1	PW	0.58
Onthophagus_curvicornis	1	Pinus	PS1	PH	0.35
Onthophagus_curvicornis	1	Pinus	PS1	EL	0.43
Onthophagus_curvicornis	1	Pinus	PS1	pTL	0.30
Onthophagus_curvicornis	1	Pinus	PS1	pTW	0.07
Onthophagus_curvicornis	1	Pinus	PS1	mTL	0.17
Onthophagus_curvicornis	1	Pinus	PS1	Biomass	0.00030
Onthophagus_curvicornis	2	Pinus	PS1	HL	0.19
Onthophagus_curvicornis	2	Pinus	PS1	HW	0.25
Onthophagus_curvicornis	2	Pinus	PS1	PL	0.35
Onthophagus_curvicornis	2	Pinus	PS1	PW	0.48
Onthophagus_curvicornis	2	Pinus	PS1	PH	0.30
Onthophagus_curvicornis	2	Pinus	PS1	EL	0.37
Onthophagus_curvicornis	2	Pinus	PS1	pTL	0.22
Onthophagus_curvicornis	2	Pinus	PS1	pTW	0.07
Onthophagus_curvicornis	2	Pinus	PS1	mTL	0.18
Onthophagus_curvicornis	2	Pinus	PS1	Biomass	0.00024
Onthophagus_curvicornis	1	Eucalyptus	ES1	HL	0.16
Onthophagus_curvicornis	1	Eucalyptus	ES1	HW	0.22
Onthophagus_curvicornis	1	Eucalyptus	ES1	PL	0.38
Onthophagus_curvicornis	1	Eucalyptus	ES1	PW	0.40
Onthophagus_curvicornis	1	Eucalyptus	ES1	PH	0.23
Onthophagus_curvicornis	1	Eucalyptus	ES1	EL	0.35
Onthophagus_curvicornis	1	Eucalyptus	ES1	pTL	0.11

Onthophagus_curvicornis	1	Eucalyptus	ES1	pTW	0.06
Onthophagus_curvicornis	1	Eucalyptus	ES1	mTL	0.13
Onthophagus_curvicornis	1	Eucalyptus	ES1	Biomass	0.00023
Onthophagus_curvicornis	2	Eucalyptus	ES1	HL	0.19
Onthophagus_curvicornis	2	Eucalyptus	ES1	HW	0.26
Onthophagus_curvicornis	2	Eucalyptus	ES1	PL	0.33
Onthophagus_curvicornis	2	Eucalyptus	ES1	PW	0.50
Onthophagus_curvicornis	2	Eucalyptus	ES1	PH	0.25
Onthophagus_curvicornis	2	Eucalyptus	ES1	EL	0.37
Onthophagus_curvicornis	2	Eucalyptus	ES1	pTL	0.23
Onthophagus_curvicornis	2	Eucalyptus	ES1	pTW	0.07
Onthophagus_curvicornis	2	Eucalyptus	ES1	mTL	0.18
Onthophagus_curvicornis	2	Eucalyptus	ES1	Biomass	0.00023
Onthophagus_curvicornis	3	Eucalyptus	ES1	HL	0.17
Onthophagus_curvicornis	3	Eucalyptus	ES1	HW	0.25
Onthophagus_curvicornis	3	Eucalyptus	ES1	PL	0.35
Onthophagus_curvicornis	3	Eucalyptus	ES1	PW	0.50
Onthophagus_curvicornis	3	Eucalyptus	ES1	PH	0.30
Onthophagus_curvicornis	3	Eucalyptus	ES1	EL	0.40
Onthophagus_curvicornis	3	Eucalyptus	ES1	pTL	0.20
Onthophagus_curvicornis	3	Eucalyptus	ES1	pTW	0.06
Onthophagus_curvicornis	3	Eucalyptus	ES1	mTL	0.18
Onthophagus_curvicornis	3	Eucalyptus	ES1	Biomass	0.00025
Onthophagus_curvicornis	4	Eucalyptus	ES1	HL	0.19
Onthophagus_curvicornis	4	Eucalyptus	ES1	HW	0.25
Onthophagus_curvicornis	4	Eucalyptus	ES1	PL	0.35
Onthophagus_curvicornis	4	Eucalyptus	ES1	PW	0.47
Onthophagus_curvicornis	4	Eucalyptus	ES1	PH	0.25
Onthophagus_curvicornis	4	Eucalyptus	ES1	EL	0.40
Onthophagus_curvicornis	4	Eucalyptus	ES1	pTL	0.21
Onthophagus_curvicornis	4	Eucalyptus	ES1	pTW	0.05
Onthophagus_curvicornis	4	Eucalyptus	ES1	mTL	0.16
Onthophagus_curvicornis	4	Eucalyptus	ES1	Biomass	0.00026
Onthophagus_curvicornis	5	Eucalyptus	ES1	HL	0.16
Onthophagus_curvicornis	5	Eucalyptus	ES1	HW	0.25
Onthophagus_curvicornis	5	Eucalyptus	ES1	PL	0.30
Onthophagus_curvicornis	5	Eucalyptus	ES1	PW	0.45
Onthophagus_curvicornis	5	Eucalyptus	ES1	PH	0.25
Onthophagus_curvicornis	5	Eucalyptus	ES1	EL	0.35
Onthophagus_curvicornis	5	Eucalyptus	ES1	pTL	0.18
Onthophagus_curvicornis	5	Eucalyptus	ES1	pTW	0.07
Onthophagus_curvicornis	5	Eucalyptus	ES1	mTL	0.15
Onthophagus_curvicornis	5	Eucalyptus	ES1	Biomass	0.00017

Onthophagus_curvicornis	1	Eucalyptus	ES2	HL	0.17
Onthophagus_curvicornis	1	Eucalyptus	ES2	HW	0.25
Onthophagus_curvicornis	1	Eucalyptus	ES2	PL	0.29
Onthophagus_curvicornis	1	Eucalyptus	ES2	PW	0.44
Onthophagus_curvicornis	1	Eucalyptus	ES2	PH	0.25
Onthophagus_curvicornis	1	Eucalyptus	ES2	EL	0.40
Onthophagus_curvicornis	1	Eucalyptus	ES2	pTL	0.20
Onthophagus_curvicornis	1	Eucalyptus	ES2	pTW	0.05
Onthophagus_curvicornis	1	Eucalyptus	ES2	mTL	0.18
Onthophagus_curvicornis	1	Eucalyptus	ES2	Biomass	0.00021
Onthophagus_curvicornis	2	Eucalyptus	ES2	HL	0.20
Onthophagus_curvicornis	2	Eucalyptus	ES2	HW	0.25
Onthophagus_curvicornis	2	Eucalyptus	ES2	PL	0.37
Onthophagus_curvicornis	2	Eucalyptus	ES2	PW	0.48
Onthophagus_curvicornis	2	Eucalyptus	ES2	PH	0.25
Onthophagus_curvicornis	2	Eucalyptus	ES2	EL	0.40
Onthophagus_curvicornis	2	Eucalyptus	ES2	pTL	0.17
Onthophagus_curvicornis	2	Eucalyptus	ES2	pTW	0.04
Onthophagus_curvicornis	2	Eucalyptus	ES2	mTL	0.15
Onthophagus_curvicornis	2	Eucalyptus	ES2	Biomass	0.00028
Onthophagus_curvicornis	3	Eucalyptus	ES2	HL	0.20
Onthophagus_curvicornis	3	Eucalyptus	ES2	HW	0.27
Onthophagus_curvicornis	3	Eucalyptus	ES2	PL	0.35
Onthophagus_curvicornis	3	Eucalyptus	ES2	PW	0.50
Onthophagus_curvicornis	3	Eucalyptus	ES2	PH	0.30
Onthophagus_curvicornis	3	Eucalyptus	ES2	EL	0.37
Onthophagus_curvicornis	3	Eucalyptus	ES2	pTL	0.16
Onthophagus_curvicornis	3	Eucalyptus	ES2	pTW	0.05
Onthophagus_curvicornis	3	Eucalyptus	ES2	mTL	0.18
Onthophagus_curvicornis	3	Eucalyptus	ES2	Biomass	0.00024
Onthophagus_curvicornis	4	Eucalyptus	ES2	HL	0.14
Onthophagus_curvicornis	4	Eucalyptus	ES2	HW	0.19
Onthophagus_curvicornis	4	Eucalyptus	ES2	PL	0.23
Onthophagus_curvicornis	4	Eucalyptus	ES2	PW	0.35
Onthophagus_curvicornis	4	Eucalyptus	ES2	PH	0.20
Onthophagus_curvicornis	4	Eucalyptus	ES2	EL	0.30
Onthophagus_curvicornis	4	Eucalyptus	ES2	pTL	0.13
Onthophagus_curvicornis	4	Eucalyptus	ES2	pTW	0.04
Onthophagus_curvicornis	4	Eucalyptus	ES2	mTL	0.11
Onthophagus_curvicornis	4	Eucalyptus	ES2	Biomass	0.00011
Onthophagus_curvicornis	5	Eucalyptus	ES2	HL	0.15
Onthophagus_curvicornis	5	Eucalyptus	ES2	HW	0.20
Onthophagus_curvicornis	5	Eucalyptus	ES2	PL	0.27

Onthophagus_curvicornis	5	Eucalyptus	ES2	PW	0.38
Onthophagus_curvicornis	5	Eucalyptus	ES2	PH	0.22
Onthophagus_curvicornis	5	Eucalyptus	ES2	EL	0.35
Onthophagus_curvicornis	5	Eucalyptus	ES2	pTL	0.15
Onthophagus_curvicornis	5	Eucalyptus	ES2	pTW	0.07
Onthophagus_curvicornis	5	Eucalyptus	ES2	mTL	0.15
Onthophagus_curvicornis	5	Eucalyptus	ES2	Biomass	0.00015
Onthophagus_curvicornis	6	Eucalyptus	ES2	HL	0.18
Onthophagus_curvicornis	6	Eucalyptus	ES2	HW	0.22
Onthophagus_curvicornis	6	Eucalyptus	ES2	PL	0.31
Onthophagus_curvicornis	6	Eucalyptus	ES2	PW	0.43
Onthophagus_curvicornis	6	Eucalyptus	ES2	PH	0.30
Onthophagus_curvicornis	6	Eucalyptus	ES2	EL	0.35
Onthophagus_curvicornis	6	Eucalyptus	ES2	pTL	0.20
Onthophagus_curvicornis	6	Eucalyptus	ES2	pTW	0.07
Onthophagus_curvicornis	6	Eucalyptus	ES2	mTL	0.16
Onthophagus_curvicornis	6	Eucalyptus	ES2	Biomass	0.00019
Onthophagus_curvicornis	7	Eucalyptus	ES2	HL	0.18
Onthophagus_curvicornis	7	Eucalyptus	ES2	HW	0.23
Onthophagus_curvicornis	7	Eucalyptus	ES2	PL	0.30
Onthophagus_curvicornis	7	Eucalyptus	ES2	PW	0.45
Onthophagus_curvicornis	7	Eucalyptus	ES2	PH	0.25
Onthophagus_curvicornis	7	Eucalyptus	ES2	EL	0.35
Onthophagus_curvicornis	7	Eucalyptus	ES2	pTL	0.15
Onthophagus_curvicornis	7	Eucalyptus	ES2	pTW	0.05
Onthophagus_curvicornis	7	Eucalyptus	ES2	mTL	0.15
Onthophagus_curvicornis	7	Eucalyptus	ES2	Biomass	0.00019
Onthophagus_curvicornis	8	Eucalyptus	ES2	HL	0.20
Onthophagus_curvicornis	8	Eucalyptus	ES2	HW	0.30
Onthophagus_curvicornis	8	Eucalyptus	ES2	PL	0.35
Onthophagus_curvicornis	8	Eucalyptus	ES2	PW	0.50
Onthophagus_curvicornis	8	Eucalyptus	ES2	PH	0.30
Onthophagus_curvicornis	8	Eucalyptus	ES2	EL	0.40
Onthophagus_curvicornis	8	Eucalyptus	ES2	pTL	0.21
Onthophagus_curvicornis	8	Eucalyptus	ES2	pTW	0.09
Onthophagus_curvicornis	8	Eucalyptus	ES2	mTL	0.18
Onthophagus_curvicornis	8	Eucalyptus	ES2	Biomass	0.00026
Onthophagus_curvicornis	9	Eucalyptus	ES2	HL	0.18
Onthophagus_curvicornis	9	Eucalyptus	ES2	HW	0.24
Onthophagus_curvicornis	9	Eucalyptus	ES2	PL	0.33
Onthophagus_curvicornis	9	Eucalyptus	ES2	PW	0.45
Onthophagus_curvicornis	9	Eucalyptus	ES2	PH	0.27
Onthophagus_curvicornis	9	Eucalyptus	ES2	EL	0.40

Onthophagus_curvicornis	9	Eucalyptus	ES2	pTL	0.17
Onthophagus_curvicornis	9	Eucalyptus	ES2	pTW	0.05
Onthophagus_curvicornis	9	Eucalyptus	ES2	mTL	0.18
Onthophagus_curvicornis	9	Eucalyptus	ES2	Biomass	0.00024
Onthophagus_curvicornis	10	Eucalyptus	ES2	HL	0.15
Onthophagus_curvicornis	10	Eucalyptus	ES2	HW	0.23
Onthophagus_curvicornis	10	Eucalyptus	ES2	PL	0.30
Onthophagus_curvicornis	10	Eucalyptus	ES2	PW	0.41
Onthophagus_curvicornis	10	Eucalyptus	ES2	PH	0.25
Onthophagus_curvicornis	10	Eucalyptus	ES2	EL	0.35
Onthophagus_curvicornis	10	Eucalyptus	ES2	pTL	0.15
Onthophagus_curvicornis	10	Eucalyptus	ES2	pTW	0.04
Onthophagus_curvicornis	10	Eucalyptus	ES2	mTL	0.15
Onthophagus_curvicornis	10	Eucalyptus	ES2	Biomass	0.00017
